# Proteome Profiling of Urinary Exosomes Identifies Alpha 1-Antitrypsin and H2B1K as Diagnostic and Prognostic Biomarkers for Urothelial Carcinoma

**DOI:** 10.1038/srep34446

**Published:** 2016-09-30

**Authors:** Shih-Yi Lin, Chao-Hsiang Chang, His-Chin Wu, Ching-Chan Lin, Kai-Po Chang, Chi-Rei Yang, Chi-Ping Huang, Wu-Huei Hsu, Chiz-Tzung Chang, Chao-Jung Chen

**Affiliations:** 1 Institute of Clinical Medical Science, China Medical University College of Medicine, Taichung, Taiwan; 2 Department of Internal Medicine, China Medical University Hospital, Taichung, Taiwan; 3 Division of Nephrology and Kidney Institute, China Medical University Hospital, Taichung, Taiwan; 4 Department of Urology, China Medical University Hospital, Taichung, Taiwan; 5 Department of Hematology and Oncology, China Medical University Hospital, Taichung, Taiwan; 6 Department of Pathology, China Medical University Hospital, Taichung, Taiwan; 7 Department of Chest Medicine, China Medical University Hospital, Taichung, Taiwan; 8 Graduate Institute of Integrated Medicine, China Medical University, Taichung, Taiwan; 9 Proteomics Core Laboratory, Department of Medical Research, China Medical University Hospital, Taichung, Taiwan

## Abstract

MALDI-TOF spectrometry has not been used for urinary exosome analysis. We used it for determining UC biomarkers. From 2012 to 2015, we enrolled 129 consecutive patients with UC and 62 participants without UC. Exosomes from their urine were isolated, and analyzed through MALDI-TOF spectrometry. Immunohistochemical (IHC) analysis of another 122 UC and 26 non-UC tissues was conducted to verify the discovered biomarkers. Two peaks at m/z 5593 (fragmented peptide of alpha-1-antitrypsin; sensitivity, 50.4%; specificity, 96.9%) and m/z 5947 (fragmented peptide of histone H2B1K sensitivity, 62.0%; specificity, 92.3%) were identified as UC diagnosis exosome biomarkers. UC patients with detectable histone H2B1K showed 2.29- and 3.11-fold increased risks of recurrence and progression, respectively, compared with those with nondetectable histone H2B1K. Verification results of IHC staining revealed significantly higher expression of alpha 1-antitrypsin (p = 0.038) and H2B1K (p = 0.005) in UC tissues than in normal tissues. The expression of alpha 1-antitrypsin and H2B1K in UC tissues was significantly correlated with UC grades (p < 0.05). Urinary exosome proteins alpha 1-antitrypsin and histone H2B1K, which are identified through MALDI-TOF analysis, could facilitate rapid diagnosis and prognosis of UC.

Urothelial carcinoma (UC), cancer of the urinary tract, is the ninth most prevalent malignancy worldwide^1^. UC is currently diagnosed through urine cytology, intravenous or computed tomography urography, and biopsy-aided cystoscopy^2^. Although urine cytology and urography are noninvasive, the UC location and grade affect the sensitivity of these tests by more than 30%^3^,^4^. Biopsy-aided cystoscopy yields the most accurate diagnosis and description of UC; however, it is expensive and invasive^5^. Thus, searching for noninvasive, objective, and rapid biomarkers that offer adequate sensitivity and specificity for the surveillance and diagnosis of UC is imperative. Recent studies have investigated the urinary proteome for UC biomarkers^6^,^7^,^8^. However, because the urinary proteome is dynamic, complex, and dependent on the biological state, highly sensitive and specific identification of UC biomarkers based on crude urine is difficult.

Exosomes are microvesicles (30–100-nm) released by cells into surrounding biofluids, including serum and urine. These vesicles participate in intercellular communication and the exchange of materials, such as proteins, RNA, and lipids^9^,^10^. Beckham *et al*. reported that urinary exosomes from patients with high-grade bladder cancer can promote UC cell migration and angiogenesis^11^. Therefore, it is believed that urinary exosomes collected from these patients may carry specific proteins for tumor development. Furthermore, exosome isolation can effectively reduce the complexity of the urinary proteome, thereby avoiding interference from highly abundant urinary proteins^12^,^13^,^14^. We developed a simple and rapid matrix-assisted laser desorption ionization time-of-flight (MALDI-TOF) platform for determining UC biomarkers that are suitable for clinical applications. We optimized the purification protocols for urinary exosomes and the MALDI-TOF platform and applied it to determine protein markers from 129 patients with and without UC. We purified and identified these protein candidates as well as immunohistochemically stained UC tissues to verify their diagnostic efficacy. The discovered biomarkers could provide a cost-effective, sensitive, and specific approach for detecting UC and predicting the risks of recurrence and progression.

## Results

### Confirmation and protein extraction of urinary microparticles

**Figure 1:**
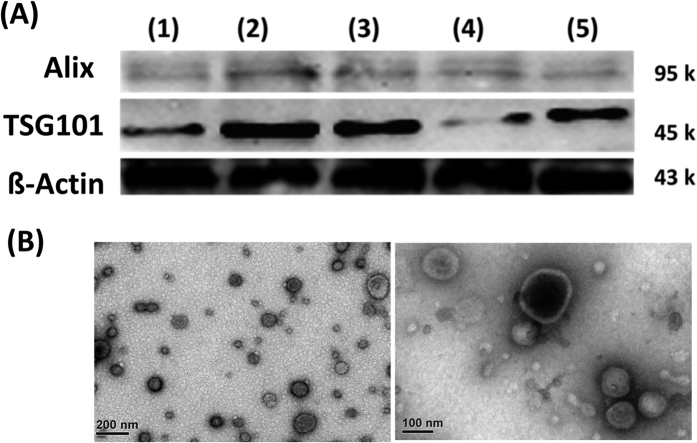
(**A**) Western blot analysis of Alix and TSG101 confirmed urinary microparticles from patients with (1) low-grade UC, (2) high-grade UC, (3) prostate cancer, (4) UTI, and (5) hernia. (**B**) Electron microscopy of urinary microparticles.

To verify successful exosome isolation through our sample preparation method, two established exosome protein markers, Alix and TSG101, were subjected to Western blotting and were detected in our samples that were isolated from the patients with UC, prostate cancer, UTI, and hernia (Fig. 1A). Electron microscopy was used to measure the size of the purified microparticles, and round membranous vesicles with approximately 50–100-nm diameters were clearly observed (Fig. 1B). Purified microparticles from the healthy participants were also subjected to protein extraction, protein digestion, and nanoLC–MS/MS analysis. A panel of known exosome markers^15^, namely mucin-1, podocalyxin, sorcin, CD9 antigen, 14-3-3 protein epsilon, elongation factor 1-alpha 1, heat shock protein 90-alpha, aldolase A, fructose bisphosphate, programmed cell death 6-interacting protein, annexin A2, glyceraldehyde-3-phosphate dehydrogenase, tumor susceptibility gene 101 protein, and aquaporin-2, was also identified in the collected samples (Supplementary Table 1).

### Comparison of different solution compositions for protein extraction from microparticles

**Figure 2:**
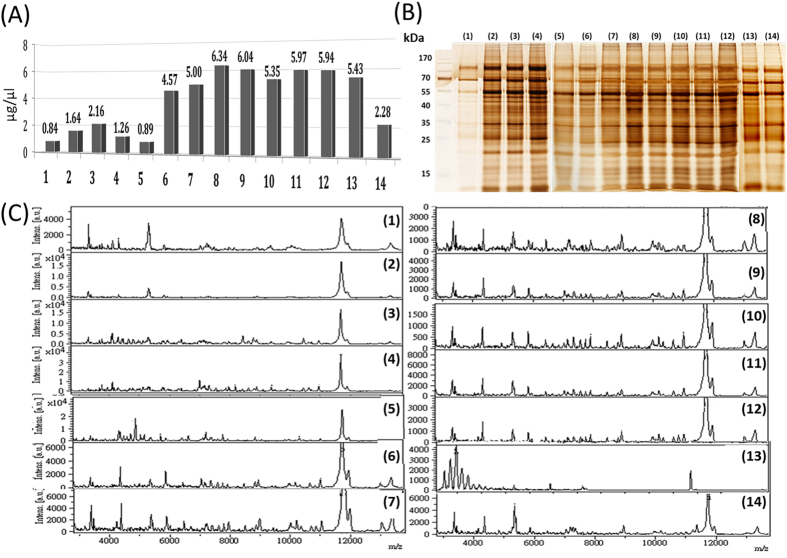
(**A**) Bradford assay, (**B**) 1D SDS–PAGE, and (**C**) MALDI-TOF analysis of proteins extracted from urinary microparticles by using different extraction solution compositions. Solution compositions are as follows: (1) ddH_2_O, (2) 25% ACN, (3) 50% ACN, (4) 75% ACN, (5) 25% FA, (6) 50% FA, (7) 75% FA, (8) 98% FA, (9) 25% FA + 50% ACN, (10) 35% FA + 50% ACN, (11) 45% FA + 50% ACN, (12) 50% FA + 50% ACN, (13) RIPA lysis buffer, and (14) sonication.

Figure 2A shows that a high percentage (75% or 98%) of FA or a mixture of 25–50% FA with 50% ACN (conditions 7–13) as the extraction solution yields a similar protein concentration, with no statistical difference (p = 0.99, ANOVA test of eight replicates at each condition) but has higher protein concentrations than do conditions 1–6 and 14. Furthermore, conditions 7–13 yield more prominent bands at both high and low molecular weights through SDS–PAGE (Fig. 2B). As expected, conditions 7–12, which yield large amounts of protein, also exhibit abundant peaks in MALDI-TOF spectra (Fig. 2C). However, because condition 13 uses RIPA lysis buffer containing detergents and salts, the MALDI-TOF signals were significantly impaired, even though the sample was purified before MALDI-TOF analysis. Therefore, 75% FA was selected as the extraction solution for subsequent experiments.

### Optimal storage conditions of urinary microparticles

The storage of clinical samples is crucial for acquiring reliable results. We evaluated suitable storage conditions for the microparticles that were isolated from one healthy participant in the form of urine, purified exosomes, and acid-extracted protein solution. Supplementary Figure 1 shows that the storage of urine or purified urinary microparticles at −80 °C for 1 and 6 mo did not affect the protein profiles. However, a prior extraction of the sample with 75% FA and long-term freezing at −80 °C significantly changed the protein profile. MALDI-TOF analysis of these frozen acid extracts of urinary exosomes revealed new complex peaks in the low m/z region, suggesting possible generation of acid-hydrolyzed peptide fragments. Therefore, we recommend purifying exosomes from fresh urine and subsequent storage at −80 °C for convenience and ensuring reliable experimental results (Supplementary Figure 2).

### Descriptive characteristics

Supplementary Table 2 shows the clinicopathological characteristics of 129 patients with UC and 62 non-UC comparison patients (25 patients with prostate cancer, 17 patients with UTI, and 20 healthy participants). No significant difference was observed between the UC and comparison groups regarding age and sex distribution (p = 0.178 and p = 0.328, respectively). In the UC group, 48.8% of the patients had pathological stage pTa/Tis/T1, 71.3% had high-grade UC, 79.9% had maximum tumor diameters of <3 cm, 62.8% had multiple tumors, 27.7% had lymphovascular invasion, 7.8% had lymph node metastasis, and 5.4% had positive surgical margins.

### Proteome profiling of urinary microparticles from UC and non-UC participants through MALDI-TOF spectrometry

**Figure 3:**
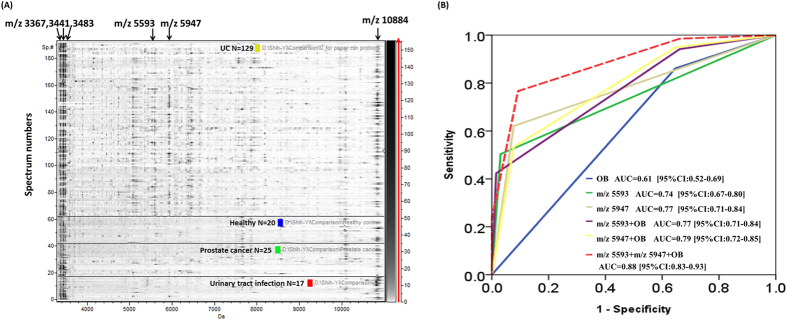
(**A**) Pseudo gels of the verification set comparing the UC (n = 129) and non-UC (prostate cancer, n = 25; UTI, n = 17; healthy, n = 20) groups. The horizontal line distinguishes between UC, healthy, prostate cancer, and UTI groups. The differential density along the x axis represents the abundance of specific peptides and proteins in the samples. (**B**) ROC curves of individual and the combination of the m/z 5593 and m/z 5947 with occult blood (+) in urine analysis.

Figure 3 provides a 2D pseudo gel comparison of the MALDI-TOF profiles of the urinary exosomes from all the UC and non-UC participants. Four peaks at m/z 3367, m/z 3441, m/z 3483, and m/z 10884 consistently appeared in the exosomes from all the participants; therefore, these peaks can act as biomarkers for identifying exosomes through MALDI-TOF spectrometry.

Two intense peaks at m/z 5593 and m/z 5947 specifically appeared in the UC group compared with the non-UC group. The sensitivity and specificity of m/z 5593 for detecting UC were 50.4% and 96.9%, respectively, with an area under the curve (AUC) of 0.736. Moreover, the sensitivity and specificity of m/z 5947 for detecting UC were 62.0% and 92.3%, respectively, with an AUC of 0.772. The sensitivity and specificity of the combination of the two peaks for detecting UC were 62.70% and 87.59%, respectively, with an AUC of 0.87.

### Comparison with the clinical method for diagnosing UC

Using a pooled sample size of 202, we compared AUC of m/z 5593 and m/z 5947 with urine analysis OB (+), the current cheapest and non-invasive clinical test for diagnosing UC in the pooled sample (n = 202). The AUC of OB, m/z 5593, and m/z 5947 were 0.61 [95%CI: 0.52–0.69], AUC = 0.74 [95%CI: 0.67–0.80], and AUC = 0.77 [95%CI:0.71–0.84], respectively (Fig. 3B). To investigate whether we could further improve the diagnosis accuracy of current clinical urinalysis, we combine urine OB (+) with m/z 5593 and m/z 5947. Overall, combination of urine OB (+), m/z 5593, and m/z 5947 yielded the largest AUC equal to 0.88 [95%CI: 0.83–0.93] (Fig. 3B).

### Association of m/z 5947 expression with UC recurrence and progression

**Figure 4:**
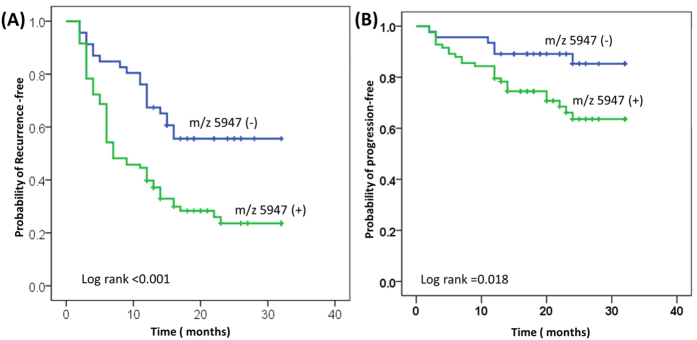
Kaplan–Meier plots of 129 patients with UC receiving TURBT, cystectomy, or nephroureterectomy, showing the probability of overall (**A**) recurrence-free and (**B**) progression-free survival, stratified by m/z 5947. TURBT: transurethral resection of bladder tumor.

The median follow-up time was 13.3 mo (2–32 mo). At the time of analysis, UC recurred in 80 of the patients (62.0%), and four of the patients died (3.1%); three of the deaths were caused by metastatic UC. In Table 1, univariate analysis indicates that m/z 5947 was associated with a higher risk of UC recurrence (hazard ratio [HR], 2.36; p = 0.001) and progression (HR, 2.77; p = 0.025). Multivariate Cox proportional hazards regression analysis indicated that m/z 5947 was an independent predictor of UC recurrence (HR, 2.29; p = 0.001) and progression (HR, 3.11; p = 0.039). Figure 4A,B depict the overall probability estimates of UC recurrence and progression.

### Purification and identification of the targeted proteins

The three exosome markers at m/z 3367, m/z 3441, and m/z 3483 were purified through 1D gradient Bis-Tris PAGE. The bands were excised, extracted, trypsin digested, and analyzed through nanoLC–MS/MS. These three markers were identified as peptide fragments of neutrophil defensin with sequences of A^31^APEQIAADIPEVVVSLAPKHPGSRKNMACYC^68^ (monoisotopic mass: m/z 3365.7; most abundant mass: m/z 3367.7), T^3^LAILAAILLVALQAQAEPQARADEVAAAPEQIA^37^ (monoisotopic mass: m/z 3439.9; most abundant mass: m/z 3440.9), and A^26^DEVAAAPEQIAADIPEVVVSLAPKHPGSRKNMA^62^ (monoisotopic mass: m/z 3481.8 Da; most abundant mass: m/z 3482.8). The UC marker peaks at m/z 5593 and m/z 5947 and the exosome marker at m/z 10884 were purified through LC, digested with trypsin, and analyzed through nanoLC–MS/MS. The m/z 5593 peak was identified as a fragment peptide of alpha 1-antitrypsin with the sequence S^307^ASLHLPKLSITGTYDLKSVLGQLGITKVFSNGADLSGVTEEAPLKLSKAVHKA^360^ (monoisotopic mass: m/z 5590.089; most abundant mass: m/z 5593.098). The m/z 5947 peak was identified as a peptide fragment of histone H2B1K (gene symbol: HIST1H2BK, UniProt accession number: O60814) with the sequence K^44^VLKQVHPDTGISSKAMGIMNSFVNDIFERIAGEASRLAHYNKRSTITSREIQ^94^ (monoisotopic mass: m/z 5944.107; most abundant mass: m/z 5947.115). The m/z 10884 peak was identified as a large peptide fragment of S100 calcium-binding protein A9 (S100A9) with the sequence R^9^NIETIINTFHQYSVKLGHPDTLNQGEFKELVRKDLQNFLKKENKNEKVIE HIMEDLDTNADKQLSFEEFI MLMARLTWAS HEKMHEGDEGP^101^ (monoisotopic mass: m/z 10878.428; most abundant mass: m/z 10884.443).

### Internal verification of alpha 1-antitrypsin and H2B1K is urinary exosomes

In split-half method, the overall predictive probability of m/z 5593 was 63.6% in the training set and 72.6% in the verification set, while the overall predictive probability of m/z 5947was 66.3% in the training set and 69.0% in the verification set. In split 1/3 method, the overall predictive probability of m/z 5593 was 74.1% in the training set and 65.3% in the verification set, while the overall predictive probability of m/z 5947 was 73.8% in the training set and 65.2% in the verification set. In both split-half and split 1/3 method, the posterior probability of m/z 5593 and m/z5947 for diagnosing UC is 100%.

### Quantitative comparison of the alpha 1-antitrypsin and H2B1K between UC and control group

Alpha 1-antitrypsin and H2B1K were identified based on peptide identifications with Mascot scores of ≥25 and a false discovery rate of <1%. The urinary exosome protein concentration of alpha 1-antitrypsin was 2.93-fold higher in UC group than in comparison group. (p = 0.001) The urinary exosome protein concentration of H2B1K was 4.85-fold higher in UC group than in comparison group (p = 0.005) (Supplementary Figure 2).

### IHC verifications of alpha 1-antitrypsin and H2B1K in tissue specimens

**Figure 5:**
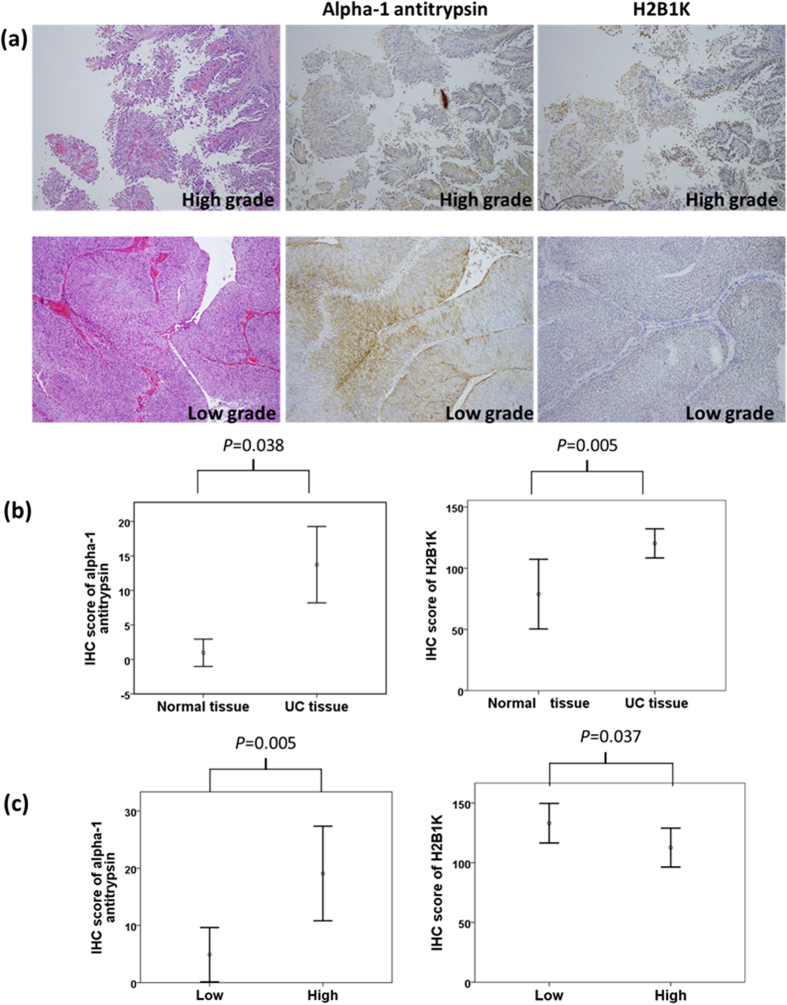
(**a**) Hematoxylin and eosin staining of high-grade (upper panel) and low-grade (low panel) UC tissues with the corresponding IHC staining of alpha 1-antitrypsin and H2B1K. Alpha 1-antitrypsin and H2B1K were subjected to cytoplasmic and nuclear staining, respectively. (**b**) Overexpression of alpha 1-antitrypsin and H2B1K in UC tissues was compared with that in normal tissues. (**c**) Different expression levels of alpha 1-antitrypsin and H2B1K in high-grade UC tissues were compared with those in low-grade UC tissues.

Figure 5a illustrates the hematoxylin and eosin staining of high- and low-grade UC tissues with the corresponding IHC staining of alpha 1-antitrypsin and H2B1K. Alpha 1-antitrypsin and H2B1K were subjected to cytoplasmic and nuclear staining, respectively. In both high- and low-grade UC, the staining intensity of alpha 1-antitrypsin and H2B1K in UC tissues was stronger than that in neighboring normal uroepithelium. We scored these IHC slides according to the staining intensity and percentage. In IHC analysis, the sensitivity and specificity of alpha 1-antitrypsin expression in UC tissue specimens were 35.2% and 96.2%, respectively, whereas those of H2B1K expression in UC tissues were 30.8% and 96.7%, respectively.

The IHC scores of alpha 1-antitrypsin expression were 4.40-fold higher in UC tissues than in normal uroepithelium (p = 0.038 and 0.005 for alpha 1-antitrypsin and H2B1K, respectively). Moreover, the IHC scores of H2B1K expression were up to 8.20-fold higher in UC tissues than in noncancerous normal tissues (Fig. 5B). Alpha 1-antitrypsin expression was 6.32-fold higher in high-grade UC tissues than in low-grade UC tissues (p = 0.005); H2B1K expression was 2.75-fold higher in low-grade UC tissues than in high-grade UC tissues (p = 0.037; Fig. 5c).

Table 2 displays the associations between the IHC scores of alpha 1-antitrypsin and H2B1K and clinicopathological characteristics. The results revealed that alpha 1-antitrypsin is significantly and positively correlated with the UC grade (p = 0.005), tumor size (p < 0.001), and UC stage (p = 0.021). Specimens from a tumor size of >3 cm had the highest IHC scores of alpha 1-antitrypsin expression, followed by those from a tumor size of 1–3 cm and <1 cm (p < 0.001). The IHC scores of alpha 1-antitrypsin expression were associated with the UC stage (p = 0.021). Stage 4 UC had the highest IHC scores of alpha 1-antitrypsin expression compared with the IHC scores of other UC stages.

## Discussion

Cancer tissues are the most suitable sources for investigating highly sensitive and specific protein biomarkers. However, obtaining tumor tissues is invasive and occasionally difficult because of anatomical limitations. Analyzing body fluids for microparticles or biomolecules secreted by tumors is a more preferable and feasible approach for discovering biomarkers because it is noninvasive, and samples can be easily obtained^16^. To our knowledge, this is the first study to use MALDI-TOF spectrometry for analyzing urinary exosomes for discovering UC biomarkers. Because urinary exosomes are rare and valuable, we first optimized protein extraction and sample preparation methods for MALDI-TOF analysis. Our results revealed that the extraction of urinary microparticles with a high-concentration FA solution or mixture of concentrated FA and ACN provides high peptide and protein extraction yields and is feasible for MALDI-TOF analysis without the detergent removal steps, which are necessary when using a detergent or salt-containing lysis buffer and could cause sample loss.

In this study, four major peaks at m/z 3367, m/z 3441, m/z 3483, and m/z 10884 were detected among exosome samples and acted as urinary exosome markers. The peaks at m/z 3367, m/z 3441, and m/z 3483 were identified as peptide fragments of neutrophil defensin, a highly basic cationic peptide that is also known as human neutrophil peptides. Neutrophil defensins are abundant in neutrophils, epithelial cells lining the bronchial tress and genitourinary tract, and urinary exosomes^17^,^18^. The m/z 10884 peak was identified as S100A9, also known as MRP-14 or calgranulin B, and has been previously identified in urinary exosomes^19^,^20^. These four urinary exosome marker peaks could provide a rapid alternative to other established tools, such as flow cytometry, western blotting, or electron microscopy, for identifying urinary exosomes.

Alpha 1-antitrypsin and H2B1K are also present in exosomes of normal urine^21^,^22^. Our study revealed that detectable m/z 5593 and m/z 5947 peaks (fragmented peptides of alpha 1-antitrypsin and histone H2B1K, respectively) in MALDI-TOF spectrometry could distinguish between UC and non-UC participants, and the AUC of the combination of m/z 5593 and m/z 5947 for diagnosing UC is >0.8. Our data suggest that the urinary exosomes of UC tissues had higher concentrations of alpha 1-antitrypsin and H2B1K than did those of non-UC tissues. In the IHC verification study, the expression of alpha 1-antitrypsin and H2B1K was 4.40- and 8.20-fold higher, respectively, in UC tissues than in noncancerous tissues. Moreover, the expression of alpha 1-antitrypsin and H2B1K in UC tissues significantly correlated with the UC grades. Alpha 1-antitrypsin and H2B1K were also observed in the secretome of bladder cancer lines and prostate cancer^21^,^22^. Altogether, alpha 1-antitrypsin and H2B1K can reasonably be determined to be UC-related proteins, which are secreted by UC tissues through exosomes into urine. The extraction of these UC urinary exosomes by performing MALDI-TOF analysis would yield significantly more abundantly detectable m/z 5593 and m/z 5947 peaks than normal urinary exosomes would. Although we have purified urinary exosomes to avoid the interferences of abundant urinary proteins, possible bias would exist in patients with renal disease such as nephrotic syndrome, glomerular disease, and chronic kidney disease which would have highly expressed levels of alpha-1antitrypsin and H2B1K in urine^23^,^24^ Biomarker potentials of alpha 1-antitrypsin and H2B1K warrant further large-scale studies for validation.

Alpha 1-antitrypsin belongs to a family of serum proteinase inhibitors. Alpha 1-antitrypsin plays a critical role in modulating immunity, inflammation, apoptosis, and possibly cellular senescence programs^25^. The absence of this multifunctional protein is associated with increased risks of lung, liver, and colorectal cancers^26^,^27^,^28^, whereas the serum level of alpha 1-antitrypsin has been reported to increase in patients with breast and prostate cancers^29^,^30^. Our IHC results revealed that alpha 1-antitrypsin expression in UC tissues had the highest positive correlation with clinical parameters of UC. Our data showed alpha 1-antitrypsin overexpression is correlated with UC grades and UC stages, which signify poor prognosis. Rajendiran *et al*. reported that miRNA-940, one of the regulators of alpha 1-antitrypsin expression, is lost in cancer tissues and cells^31^. They revealed that miRNA-940 can inhibit the migratory and invasive potential of cells, attenuate their anchorage-independent growth ability, increase E-cadherin expression, and suppress prostate cancer progression^31^. Together, their observations and our results support that alpha 1-antitrypsin can be used as a potential biomarker for UC diagnosis and probable prognosis. In our study, the m/z 5947 peak in urine exosomes was associated with 2.29- and 3.11-fold increased risks of UC recurrence and progression, respectively. Certain clinical and pathological factors, such as the pathological grade, tumor size, pathological staging, surgical margin, lymph node status, lymphovascular invasion, and age are of prognostic value in predicting UC recurrence and progression^32^. After adjusting for these factors, we determined that m/z 5947 remains an independent predictor of UC recurrence and progression. Notably, our IHC results revealed that H2B1K staining was stronger in low-grade UC tissues than in high-grade UC tissues. Histone H2B is one of the most abundantly monoubiquitinated conjugates in the nucleus and is involved in the transcriptional control of gene expression and the DNA damage response^33^. Levels of ubiquitinated H2B are low in advanced cancers, including breast, colorectal, lung, and parathyroid cancers^34^. The modification patterns of histones are functionally associated with the transcriptional activity of tumor suppressor genes^35^. Inhibiting deubiquitinase is a novel strategy for developing cancer therapeutics^36^. The alteration of cell cycle kinetics, which involves histone modification^37^, is typically the hallmark of high-grade or aggressive cancers^38^. Loddol *et al*. reported that the cell cycle phenotype, including the arrest of differentiation and increasing genomic instability, is correlated with aggressive tumors and disease progression^38^. Bonenfant *et al*. observed dynamic changes in histone modification for parallel periods of the cell cycle^39^.

Thus, one possible explanation for our lower H2B1K expression in high-grade UC tissues is that the disease progression potential was majorly determined by histone modification levels rather than histone expression levels.

To date, few potential markers, such as cadherin-1^40^, hypoxia-inducible factor 1α^41^, survivin^42^, and C-reactive protein^43^, have been reported as independent predictors of UC recurrence and survival. Lotan *et al*. used a panel of molecular markers, namely p53, p21, p27, ki-67, and cyclin-E1, to predict recurrence and cancer-specific survival after radical cystectomy^44^. However, these markers were detected in tissues and blood, specimens of invasive approaches, which are more difficult to directly translate into clinical applications.

The m/z 5947 peak of urinary UC exosomes identified through MALDI-TOF spectrometry provides a rapid and noninvasive strategy for direct translation into clinical practice. Furthermore, our participants represent all UC characteristics, including the tumor location, treatment, and pathological grades, thereby reflecting an actual scenario that might avoid possible selection bias, such as unequal recurrence rates among different locations and treatments, and providing a more applicable biomarker candidate for diagnosing and predicting UC.

In summary, m/z 3367, m/z 3441, m/z 3483, and m/z 10884 were identified as markers for detecting urinary exosomes. These findings would facilitate rapid examination and optimization of exosome purification efficiency. The m/z 5593 and m/z 5947 peaks in urinary exosomes were identified as biomarkers for UC. The UC patients with the m/z 5947 peak had 2.29- and 3.11-fold increased risks of UC recurrence and progression, respectively. In addition, the overexpression of alpha 1-antitrypsin and H2B1K was reported in UC tissues. Our paper provides a potential rapid and analytical platform for discovering urinary exosomes and biomarkers as well as warrants further large-scale validation studies.

## Materials and Methods

### Experimental design

The objective of this study was to explore the presence of peaks (peptide fragment) in urine analyzed by MALDI-TOF spectrometry that could serve as biomarkers for UC. We first determine the appropriate methods for exosome protein extraction and appropriate conditions for MALDI-TOF analysis. We consecutively collected urine samples of 129 UC patients and 62 controls. Urinary exosomes were separated, of which exosomes proteins were extracted, and analyzed with MALDI-TOF spectrometry. In parallel, the classical clinical data were obtained including age, gender, cancer staging, cancer grading, recurrence, and disease progression. The candidate peaks of which are under curve of ROC curve >0.7 were chosen for UC biomarkers. The candidate peaks were purified. Verification of these proteins for UC biomarker was carried out in two ways: internal validation of pooled exosomes samples (n = 202), and immunohistochemical stating of 122 UC tissues and 26 normal uroepithelial tissues.

### Clinical sample collection and processing

The human research protocols were approved by the Medical Ethics and Human Clinical Trial Committee of China Medical University Hospital, Taiwan. Informed consent was obtained from all subjects. All methods were carried out in accordance with the relevant ethic guidelines. First morning urine samples were collected from patients with hernia, urinary tract infection (UTI), prostate cancer, or UC. A protease inhibitor cocktail tablet (Roche, Mannheim, Germany) was added in each urine specimen (approximately 50 mL). The specimens were then centrifuged at 1000× *g* for 10 min to remove debris and stored at −80 °C until the subsequent purification of urinary microparticles, and clinicpathological variables were analyzed. Disease progression was defined as distant metastasis, superficial progression to muscle invasion, or cancer-related death. Recurrence was defined as a new tumor developed after the transurethral resection of a bladder tumor, secondary primaries, progression, or distant metastasis. To confirm our discovered biomarkers expressed differently between UC and controls, iTRAQ labelling quantitative nanoLC–MS/MS was carried out for UC (n = 5) and non-UC (n = 10) groups.

To confirm and validate our discovered biomarkers, we categorized another set of participants into the UC (n = 122) and non-UC (n = 26) groups. Surgical specimens of their UC and non-UC tissues were analyzed through immunohistochemical (IHC) staining of alpha 1-antitrypsin and H2B1K.

### Isolation of urinary microparticles

Urinary microparticles were prepared through ultracentrifugation, as previously described^45^,^46^. The standard protocol for isolating these microparticles is provided in Supplementary Figure 3. Urine (50 mL) was centrifuged at 17000× *g* for 10 min at 4 °C (Ti70 rotor; Beckman Coulter AB, Bromma, Sweden); the supernatant was collected as SN1. The pellets were resuspended in an isolation solution (10 mm triethanolamine, 250 mm sucrose, pH 7.6, 0.5 mm phenylmethanesulfonyl fluoride) before 200 mg/mL dithiothreitol was added and before incubation at 95 °C for 2 min. The resuspended solution was centrifuged at 17000× *g* for 30 min at 4 °C, and the supernatant was collected as SN2. SN1 and SN2 were pooled and ultracentrifuged at 200000× *g* for 1 h at 4 °C. The supernatant was removed, and the microparticles were collected for further analysis.

### Western immunoblotting

The microparticles were harvested using an RIPA lysis buffer, and 20 μg of proteins was solubilized in Laemmli sample buffer (1.5% sodium dodecyl sulfate [SDS], 6% glycerol, and 10 mm Tris-HCl, pH 6.8). Proteins were separated through one-dimensional (1D) SDS–polyacrylamide gel electrophoresis (SDS–PAGE) and electrophoretically transferred onto polyvinylidene fluoride (PVDF) membranes. After the PVDF membrane was blocked with 5% nonfat milk at room temperature for 1 h, the membranes were probed overnight at 4 °C with primary monoclonal antibodies to TSG101 (1:500; Thermo Scientific, Rockford, USA), Alix (1:200; Chemicon, Germany), or actin (1:1000; Rockland, PA, USA). Thereafter, the membranes were probed with peroxidase-conjugated antirabbit or antimouse IgG (1:10000) for 1 h at room temperature. Immunoreactivity was detected through enhanced chemiluminescence.

### Transmission electron microscopic analysis

The microparticles were resuspended in 2.5% glutaraldehyde and applied to Formvar-coated carbon-stabilized copper grids. The grids were dried at room temperature, washed twice with phosphate-buffered saline, and then stained with 2% (w/v) uranyl acetate for 10 min. The grid was examined using a JEOL 200CX electron microscope.

### SDS–PAGE

The extracted proteins were separated on 12% SDS polyacrylamide gels. Electrophoresis was performed using a power supply set at 75 V/gel and 110 V/gel for the stacking and resolving gels, respectively. All the gels were placed in a fixing solution (methanol:acetic acid:deionized water = 40:10:50) for 1 h, washed twice in ddH_2_O for 10 min each, and stained with silver stain.

### Silver stain

The gels were first fixed in 50% methanol and 5% acetic acid in ddH_2_O for 20 min. The gels were rinsed twice in 30% ethanol for 10 min and twice in ddH_2_O for 10 min. The gels were then incubated in 0.02% sodium thiosulfate for 1 min and rinsed twice with ddH_2_O for 1 min each. Furthermore, the gels were placed in 0.1% silver nitrate solution for 20 min, rinsed twice with ddH_2_O for 1 min each, and developed in 0.04% formalin (35% formaldehyde) in 2% sodium carbonate with constant shaking. The development was terminated by adding 5% acetic acid after achieving the desired intensity of silver staining.

### Solution compositions for protein extraction of microparticles

Totally, 14 different extraction solutions were tested: (1) ddH2O, (2) 25% acetonitrile (ACN), (3) 50% ACN, (4) 75% ACN, (5) 25% formic acid (FA), (6) 50% FA, (7) 75% FA, (8) 98% FA, (9) 25% FA + 50% ACN, (10) 35% FA + 50% ACN, (11) 45% FA + 50% ACN, (12) 50% FA + 50% ACN, (13) RIPA buffer sonication, and (14) sonication. The RIPA buffer contained 10 mM Tris-HCl, 1 mM ethylenediaminetetraacetic acid, 1 mM ethylene glycol tetraacetic acid, 50 mM NaCl, 50 mM NaF, 20 mM Na_4_P_2_O_7_, 1 mM Na_3_VO_4_, and 1% Triton X-100. Sonication was performed for 100 s at 180 W with alternating 10-s sonication–rest for each cycle. After extraction, the insoluble sediment was removed through centrifugation at 10000× *g* for 15 min at 4 °C. The protein concentration of each method was measured using the Bradford assay at 562 nm. This extraction protocol was repeated for eight biological replicates (Supplementary Figure 4).

### Assessing the effects of the storage status

The first morning urine sample was collected from one participant. The microparticles were isolated from one fraction of the fresh urine, extracted with 75% FA solution, and immediately analyzed through MALDI-TOF spectrometry to determine the urinary exosome protein profile without storage. Furthermore, the microparticles were isolated from the remaining fraction of the urine sample, extracted with 75% FA peptide–protein solution, and stored for a long term at −80 °C. Analysis was performed after 1 and 6 mo through MALDI-TOF spectrometry.

### MALDI-TOF analysis

The analyte solution was mixed with saturated sinapinic acid (SA) solution (30:70 ACN:0.1% trifluoroacetic acid) at volume ratios of 1:1 or 1:5. One microliter of the analyte/SA solution was placed on the MALDI-TOF target. After analyte/SA cocrystallization, the sample plate was analyzed through MALDI-TOF spectrometry (Ultraflex III TOF/TOF; Bruker Daltonics). MALDI-TOF spectrometry was operated in a linear positive ion mode with 25-kV accelerating voltage at a laser frequency of 50 Hz with a mass range of 1000–23000 Da. Peptide–protein calibrations were conducted using a peptide–protein calibration standard kit (Bruker Daltonics). MALDI-TOF mass spectra were processed using flexAnalysis 3.0 software (Bruker Daltonics).

### Purification and identification of peptide and protein marker peaks

For higher separation resolution, electrophoresis on 4–12% Bis-Tris NuPAGE® gels (Invitrogen, San Diego, CA, USA) was performed to purify the marker peaks at m/z 3367, m/z 3441, and m/z 3483. Electrophoresis was performed at 75 V/gel and 110 V/gel for the stacking and resolving gels, respectively. After the separation, the gels were stained with Coomassie brilliant blue G250 and destained using ddH_2_O. Peptide bands of approximately 3000 Da were excised and from both exosome extracts and control sample (blood added into ddH2O to avoid interference of urinary proteins), extracted using 50% ACN/0.1% FA. The extracted peptides were analyzed through MALDI-TOF spectrometry to confirm the presence of peaks at m/z 3367, m/z 3441, and m/z 3483. The extracted peptides were then reduced, alkylated, trypsin digested, and finally dried for further peptide identification through nanoflow liquid chromatography (nanoLC)–tandem mass spectrometry (MS)/MS analysis. The peak identification using Bis-Tris gels has been performed with three biological replicates. Each replicate resulted from exosome extracts can have the same marker peaks at m/z 3367, m/z 3441, and m/z 3483 on MALDI–TOF (Supplementary Figure 5).

An LC pumping system (Ultimate 3000, Dionex, The Netherlands) equipped with an LC column (C-4 bonded particles, 2.1 mm × 250 mm, Walters) was used for protein purification. The mobile phases were solvent A (5% ACN and 0.1% FA) and solvent B (100% ACN and 0.1% FA). Gradient elution at a flow rate of 250 μL/min was set as follows: 0% B for 10 min, 15–80% B for 12 min, and 80–100% B for 3 min. The eluents were monitored using a UV detector (VWD-3400 RS, Dionex, The Netherlands) at wavelengths of 210 nm and 280 nm. The eluents were collected at 30-s intervals. Each fraction was analyzed through MALDI-TOF spectrometry to confirm the successful purification of the marker peaks at m/z 5593, m/z 5947, and m/z 10 884. The purified peptides and proteins were subjected to in-solution digestion and nanoLC–MS/MS analysis for identification.

### Protein Purification and iTRAQ Sample Labelling

Proteins extract of 100 μg each sample was purified as following: added with four times the volume of cold (−20 °C) acetone containing 12% (w/v) TCA, the mixture was incubated 6 hours at −20 °C, centrifuged at 12,000× g at 4 °C for 30 minutes, and then the supernatant was removed without disturbing the visible pellet. Four times the sample volume of cold (−20 °C) acetone was added to the pellet. After incubation for 4 hours at −20 °C and centrifugation at 12,000× g at 4 °C for 30 minutes, the supernatant was removed and the pellet air dried. The protein pellets were then dissolved in the solution buffer, reduced, and blocked with cysteins, as as suggested by the manufacturers. Each sample was trypsin digested (2%, w/w) at 37 °C overnight and then labeled with the iTRAQ tags separately as UC or controls. The labeled digests were then mixed and dried for further quantitative nanoLC–MS/MS analysis.

### NanoLC–MS/MS analysis

NanoLC–MS/MS was performed using a nanoflow ultra performance liquid chromatography system (UltiMate 3000 RSLCnano system; Dionex, The Netherlands) coupled to a hybrid quadrupole TOF (Q-TOF) mass spectrometer (maXis impact; Bruker Daltonics). Tryptic peptide mixtures were injected using an autosampler and loaded at a flow rate of 15 μL/min on a self-packed C18 trap column (180 μm i.d.; length, 2 cm; particle size, 5 μm) for desalting and preconcentration for 5 min. The peptides were then eluted into an analytical column (Acclaim PepMap C18, 2 μm, 100 Å, 75 μm × 250 mm, Thermo Scientific, USA) coupled to a nanoelectrospray ionization source on the Q-TOF mass spectrometer. A gradient elution of 1% ACN (0.1% FA) to 40% ACN (0.1% FA) for 90 min was conducted at a flow rate of 300 nL/min for peptide separation. Ten precursors of charge +2, +3, and +4 from each TOF-MS scan (m/z 50–2000) were dynamically selected and isolated for MS/MS fragment ion scanning (m/z 50–2000). The selected precursors were then actively excluded for 25 s. MS and MS/MS accumulation were set at 1 and 10 Hz, respectively.

### IHC staining

Tissue specimens were initially formalin fixed and paraffin embedded. All IHC staining was conducted using a Leica Bond-Max autostainer (Leica Microsystems) according to the manufacturer’s protocol. Antigen retrieval was conducted at pH 8 by using Epitope Retrieval 2 solution (Leica Microsystems) for 20 min at 100 °C. The slides were then incubated for 15 min at room temperature with primary antibodies at the following dilutions: rabbit polyclonal antitrypsin (1:1600; Novocastra) and rabbit polyclonal H2B1K (1:400; NOVUS). A polymer detection system (Bond Polymer Refine; Vision BioSystems) was used.

The slides were examined by a pathologist who was blinded to the outcomes and disease status of the participants. The proportion score described the estimated fraction of positive stained tumor tissues (0–100%). The intensity score represented the estimated staining intensity (0, no staining; 1, weak; 2, moderate; 3, strong). The total score, the proportion score multiplied by the intensity score, ranged from 0 to 300.

### Statistical analysis

Statistical analysis of the MS profiling spectra from the UC and non-UC participants (patients with prostate cancer and UTI and healthy participants) was performed using ClinPro Tools software (Version 3.0, Bruker Daltonics). Univariate and multivariate Cox regression analysis was used to determine the association between different clinicopathological variables and biomarkers and disease recurrence and progression. All the spectra were normalized to their own total ion count. The MS data from all the participants are presented in a two-dimensional (2D) cluster plot. A list of peaks with statistical differences between two classes that were determined using a t test and analysis of variance (ANOVA) were selected. We combined MALDI-spectra of 126 exosomes and 54 exosomes enrolled later to be a pooled sample (n = 202; UC = 137, comparison = 65). Split-sample validation was done in this pooled sample. We adapted split-half and split 1/3 method, where 50% or 33.33% of the sample were kept as an independent evaluation part for the m/z 5593 and m/z 5947 that was estimated on 50% or 66.67% of the sample, respectively. The split was made once and at random.

The Mann–Whitney U test was used to analyze the associations between the IHC scores of the candidate proteins and different clinical parameters. Two-tailed p values of ≤0.05 were considered significant. The statistical package SPSS 20.0 (SPSS, Inc., Chicago, IL) was used to analyze all the clinical data.

## Additional Information

**How to cite this article**: Lin, S.-Y. *et al*. Proteome Profiling of Urinary Exosomes Identifies Alpha 1-Antitrypsin and H2B1K as Diagnostic and Prognostic Biomarkers for Urothelial Carcinoma. *Sci. Rep.* **6**, 34446; doi: 10.1038/srep34446 (2016).

## Supplementary Material

Supplementary Information

## Figures and Tables

**Table 1:** Univariate and multivariate Cox regression analysis predicts disease recurrence and progression in patients with UC.

Predictors	Recurrence				Progression			
	Univariate analysis		Multivariate analysis		Univariate analysis		Multivariate analysis	
	HR	P	HR	P	HR	P	HR	P
Age (≧65 yrs vs ≦65 yrs)	1.79	0.021	1.71	0.034**	2.35	0.046	2.35	0.096
Gender: males vs female	1.003	0.99	1.089	0.722	1.048	0.899	1.036	0.481
Tumor size								
1–3 cm vs <1 cm	1.194	0.535	1.31	0.371	5.59	0.02	5.15	0.031
≧3 cm vs <1 cm	1.022	0.933	1.11	0.794	4.67	0.05	1.93	0.46
Grade: High vs Low	1.08	0.754	1.03	0.909	2.63	0.027	2.53	0.034
Stage								
T2 vs Tis/Ta/T1	1.52	0.157	1.346	0.3	1.08	0.867	1.07	0.893
T3/T4 vs T2	1.72	0.75	1.249	0.41	1.18	0.686	1.17	0.385
Numbers of tumors: multiple vs single	1.10	0.678	1.10	0.644	5.58	0.02	1.568	0.367
Lymph node status: metastatsis	2.058	0.16	1.923	0.206	1.23	0.776	1.079	0.47
Lymphovascular invasion: positive	1.196	0.609	1.017	0.963	1.71	0.46	1.58	0.46
Chemotherapy*: Yes, no	1.16	0.501	1.01	0.965	1.54	0.227	1.42	0.338
Surgical margin: positive vs negative	1.37	0.493	1.14	0.774	7.58	<0.001	4.13	**0.031**
m/z 5947: positive	2.36	0.001	2.29	0.001**	2.77	0.025	3.11	0.039**
m/z 5593: positive	1.54	0.647	1.27	0.289	1.156	0.683	1.222	0.649

HR: Hazard ratio; pT, pathological tumor; *Chemotherapy includes intravesical treatment and neoadjuvant chemotherapy **p < 0.05.

**Table 2:** Correlations of IHC staining score of alpha-1 antitrypsin and H2B1K with clinical parameters.

IHC staining	Normal versus UC			p value	
	Normal (26)	UC (122)			
Alpha-1-antirtypsin	55.48	78.55		0.002*	
H2B1K	54.13	78.84		0.007*	
Age					
	^<^65 (19)	≥65 (103)			
Alpha-1-antirtypsin	54.89	62.72		0.299	
H2B1K	57.47	62.24		0.587	
Grade					
	Low (46)	High (76)			
Alpha-1-antirtypsin	51.73	67.41		0.005*	
H2B1K	70.01	56.35		0.037*	
Tumor size					
	<1 cm (61)	1–3 cm (43)	≥3 cm (18)		
Alpha-1-antirtypsin	53.01	60.78	92	<0.001*	
H2B1K	62.28	62.29	56.03	0.773	
Number of tumors					
	Single (72)	Multiple (49)			
Alpha-1-antirtypsin	64.41	55.99		0.129	
H2B1K	61.17	60.74		0.947	
T					
	Ta + Tis	T1 + T2	T3 + T4		
Alpha-1-antirtypsin	49.88	62.94	61.48	0.512	
H2B1K	94.25	56.13	63.43	0.013*	
N					
	N0 (117)	N1 (2)	N2 (2)		
Alpha-1-antirtypsin	59.97	70.25	111.75	0.046	
H2B1K	60.75	70.5	66.0	0.907	
M					
	M0 (117)	M1 (5)			
Alpha-1-antirtypsin	60.43	86.6		0.057	
H2B1K	62.9	28.7		0.033*	
Stage					
	0 (7)	I + II (64)	III (38)	IV (13)	
Alpha-1-antirtypsin	51.29	61.13	56.07	84.69	0.021*
H2B1K	95.64	58.06	62.01	58.54	0.062
