# Supplementary material for: Proteome Profiling of Urinary Exosomes Identifies Alpha 1-Antitrypsin and H2B1K as Diagnostic and Prognostic Biomarkers for Urothelial Carcinoma
Source: Sci Rep. 2016 Sep 30;6:34446. doi: 10.1038/srep34446 (PMC5043375; doi:10.1038/srep34446)
Supplement: Supplementary Information [file srep34446-s1.pdf]

Proteome Profiling of Urinary Exosomes Identifies Alpha 1-Antitrypsin and H2B1K as Diagnostic and Prognostic Biomarkers for Urothelial Carcinoma

Shih-Yi Lin, MD [a,b,c], Chao-Hsiang Chang [d], His-Chin Wu[d], Ching-Chan Lin[a,e], Kai-Po Chang[f], Chi-Rei Yang[d], Chi-Ping Huang[a,d], Wu-Huei Hsu[a,b,g], Chiz-Tzung Chang [a,b,c], Chao-Jung Chen[h,i]

[a]Institute of Clinical Medical Science, China Medical University College of Medicine, Taichung, Taiwan
[b]Department of Internal Medicine, China Medical University Hospital, Taichung, Taiwan
[c]Division of Nephrology and Kidney Institute, China Medical University Hospital, Taichung, Taiwan
[d]Department of Urology, China Medical University Hospital, Taichung, Taiwan
[e]Department of Hematology, China Medical University Hospital, Taichung, Taiwan
[f] Department of Pathology, China Medical University Hospital, Taichung, Taiwan
[g]Department of Chest Medicine, China Medical University Hospital, Taichung, Taiwan
[h]Graduate Institute of Integrated Medicine, China Medical University, Taichung, Taiwan
[i]Proteomics Core Laboratory, Department of Medical Research, China Medical University Hospital, Taichung, Taiwan

Corresponding author*
Chao-Jung Chen, Ph.D.
Associate Professor
Graduate institute of integrated medicine
China Medical University
91, Hsueh-Shih Rd, Taichung, Taiwan, R.O.C
E-mail: cjchen@mail.cmu.edu.tw
Tel: 886-4-22052121
Keywords: Exosome biomarker, Urothelial carcinoma, Matrix-assisted laser desorption/ionization time-of-flight mass spectrometry, Prognostic

Supplementary table 1　List of identified urinary exosomes protein

Accession	Protein	MW [kDa]	pI	Scores	SC [%]
ZBT45_HUMAN	Zinc finger and BTB domain-containing protein 45 OS=Homo sapiens GN=ZBTB45 PE=2 SV=1	54.0	6.5	32.0 (M:32.0)	1.4
ZA2G_HUMAN	Zinc-alpha-2-glycoprotein OS=Homo sapiens GN=AZGP1 PE=1 SV=2	34.2	5.7	228.6 (M:228.6)	19.8
YIPF3_HUMAN	Protein YIPF3 OS=Homo sapiens GN=YIPF3 PE=1 SV=1	38.2	5.4	28.8 (M:28.8)	2.9
VTNC_HUMAN	Vitronectin OS=Homo sapiens GN=VTN PE=1 SV=1	54.3	5.5	400.0 (M:400.0)	12.1
VPS4A_HUMAN	Vacuolar protein sorting-associated protein 4A OS=Homo sapiens GN=VPS4A PE=1 SV=1	48.9	8.6	77.7 (M:77.7)	5.3
VMO1_HUMAN	Vitelline membrane outer layer protein 1 homolog OS=Homo sapiens GN=VMO1 PE=1 SV=1	21.5	4.8	53.1 (M:53.1)	5.9
VIME_HUMAN	Vimentin OS=Homo sapiens GN=VIM PE=1 SV=4	53.6	4.9	55.6 (M:55.6)	4.1
VDAC2_HUMAN	Voltage-dependent anion-selective channel protein 2 OS=Homo sapiens GN=VDAC2 PE=1 SV=2	31.5	8.7	50.3 (M:50.3)	4.1
VDAC1_HUMAN	Voltage-dependent anion-selective channel protein 1 OS=Homo sapiens GN=VDAC1 PE=1 SV=2	30.8	9.2	207.0 (M:207.0)	18.7
VATE1_HUMAN	V-type proton ATPase subunit E 1 OS=Homo sapiens GN=ATP6V1E1 PE=1 SV=1	26.1	8.8	54.7 (M:54.7)	4.9
VATB1_HUMAN	V-type proton ATPase subunit B, kidney isoform OS=Homo sapiens GN=ATP6V1B1 PE=1 SV=3	56.8	5.3	56.1 (M:56.1)	4.3
VATA_HUMAN	V-type proton ATPase catalytic subunit A OS=Homo sapiens GN=ATP6V1A PE=1 SV=2	68.3	5.2	109.6 (M:109.6)	4.7
VAT1_HUMAN	Synaptic vesicle membrane protein VAT-1 homolog OS=Homo sapiens GN=VAT1 PE=1 SV=2	41.9	5.9	35.8 (M:35.8)	2.5
UTER_HUMAN	Uteroglobin OS=Homo sapiens GN=SCGB1A1 PE=1 SV=1	10.0	4.8	48.8 (M:48.8)	11.0
UROM_HUMAN	Uromodulin OS=Homo sapiens GN=UMOD PE=1 SV=1	69.7	4.9	44663.8 (M:44663.8)	46.7
UPK3A_HUMAN	Uroplakin-3a OS=Homo sapiens GN=UPK3A PE=1 SV=3	30.7	4.5	51.3 (M:51.3)	8.0
UPK1A_HUMAN	Uroplakin-1a OS=Homo sapiens GN=UPK1A PE=2 SV=1	28.9	5.0	45.9 (M:45.9)	8.9
TTHY_HUMAN	Transthyretin OS=Homo sapiens GN=TTR PE=1 SV=1	15.9	5.4	481.5 (M:481.5)	33.3
TSN1_HUMAN	Tetraspanin-1 OS=Homo sapiens GN=TSPAN1 PE=1 SV=2	26.3	5.0	265.3 (M:265.3)	5.4
TS101_HUMAN	Tumor susceptibility gene 101 protein OS=Homo sapiens GN=TSG101 PE=1 SV=2	43.9	6.0	41.6 (M:41.6)	2.6
TRFL_HUMAN	Lactotransferrin OS=Homo sapiens GN=LTF PE=1 SV=6	78.1	9.6	1155.2 (M:1155.2)	38.3
TRFE_HUMAN	Serotransferrin OS=Homo sapiens GN=TF PE=1 SV=3	77.0	7.0	389.9 (M:389.9)	9.5
TPP1_HUMAN	Tripeptidyl-peptidase 1 OS=Homo sapiens GN=TPP1 PE=1	61.2	6.0	203.5	7.5

	SV=2			(M:203.5)	
TPIS_HUMAN	Triosephosphate isomerase OS=Homo sapiens GN=TPI1 PE=1 SV=3	30.8	5.6	293.6 (M:293.6)	28.3
TM256_HUMAN	Transmembrane protein 256 OS=Homo sapiens GN=TMEM256 PE=2 SV=1	11.7	9.9	145.2 (M:145.2)	24.8
TIMP1_HUMAN	Metalloproteinase inhibitor 1 OS=Homo sapiens GN=TIMP1 PE=1 SV=1	23.2	9.8	70.2 (M:70.2)	17.9
THTM_HUMAN	3-mercaptopyruvate sulfurtransferase OS=Homo sapiens GN=MPST PE=1 SV=3	33.2	6.1	218.6 (M:218.6)	18.9
THRB_HUMAN	Prothrombin OS=Homo sapiens GN=F2 PE=1 SV=2	70.0	5.6	135.7 (M:135.7)	6.3
THIL_HUMAN	Acetyl-CoA acetyltransferase, mitochondrial OS=Homo sapiens GN=ACAT1 PE=1 SV=1	45.2	9.6	93.5 (M:93.5)	5.9
TENX_HUMAN	Tenascin-X OS=Homo sapiens GN=TNXB PE=1 SV=3	464.0	5.1	70.4 (M:70.4)	0.2
TBB4A_HUMAN	Tubulin beta-4A chain OS=Homo sapiens GN=TUBB4A PE=1 SV=2	49.6	4.6	165.5 (M:165.5)	8.1
TBA1A_HUMAN	Tubulin alpha-1A chain OS=Homo sapiens GN=TUBA1A PE=1 SV=1	50.1	4.8	96.8 (M:96.8)	3.3
SYPL1_HUMAN	Synaptophysin-like protein 1 OS=Homo sapiens GN=SYPL1 PE=1 SV=1	28.5	9.5	47.8 (M:47.8)	4.2
SUSD2_HUMAN	Sushi domain-containing protein 2 OS=Homo sapiens GN=SUSD2 PE=1 SV=1	90.1	5.8	40.3 (M:40.3)	5.1
STOM_HUMAN	Erythrocyte band 7 integral membrane protein OS=Homo sapiens GN=STOM PE=1 SV=3	31.7	8.9	176.5 (M:176.5)	16.7
SPIT3_HUMAN	Kunitz-type protease inhibitor 3 OS=Homo sapiens GN=SPINT3 PE=2 SV=3	10.2	7.2	50.2 (M:50.2)	19.1
SPEB_HUMAN	Agmatinase, mitochondrial OS=Homo sapiens GN=AGMAT PE=1 SV=2	37.6	8.9	76.0 (M:76.0)	14.2
SORCN_HUMAN	Sorcin OS=Homo sapiens GN=SRI PE=1 SV=1	21.7	5.2	63.5 (M:63.5)	8.1
SODM_HUMAN	Superoxide dismutase [Mn], mitochondrial OS=Homo sapiens GN=SOD2 PE=1 SV=2	24.7	9.1	84.9 (M:84.9)	10.4
SODC_HUMAN	Superoxide dismutase [Cu-Zn] OS=Homo sapiens GN=SOD1 PE=1 SV=2	15.9	5.7	52.8 (M:52.8)	9.1
SLPI_HUMAN	Antileukoproteinase OS=Homo sapiens GN=SLPI PE=1 SV=2	14.3	10.7	50.0 (M:50.0)	9.1
SEMG2_HUMAN	Semenogelin-2 OS=Homo sapiens GN=SEMG2 PE=1 SV=1	65.4	9.6	4842.5 (M:4842.5)	48.1
SEMG1_HUMAN	Semenogelin-1 OS=Homo sapiens GN=SEMG1 PE=1 SV=2	52.1	9.7	4545.0 (M:4545.0)	48.1
SDCB1_HUMAN	Syntenin-1 OS=Homo sapiens GN=SDCBP PE=1 SV=1	32.4	7.9	339.6 (M:339.6)	22.8
SDC4_HUMAN	Syndecan-4 OS=Homo sapiens GN=SDC4 PE=1 SV=2	21.6	4.2	193.3 (M:193.3)	12.1
SDC1_HUMAN	Syndecan-1 OS=Homo sapiens GN=SDC1 PE=1 SV=3	32.4	4.4	141.0 (M:141.0)	5.5

SCTM1_HUMAN	Secreted and transmembrane protein 1 OS=Homo sapiens GN=SECTM1 PE=1 SV=2	27.0	7.9	108.9 (M:108.9)	14.1
SC5AA_HUMAN	Sodium/glucose cotransporter 5 OS=Homo sapiens GN=SLC5A10 PE=1 SV=2	64.3	8.7	142.6 (M:142.6)	4.2
SAP_HUMAN	Proactivator polypeptide OS=Homo sapiens GN=PSAP PE=1 SV=2	58.1	4.9	217.8 (M:217.8)	6.7
SAP3_HUMAN	Ganglioside GM2 activator OS=Homo sapiens GN=GM2A PE=1 SV=4	20.8	5.0	200.5 (M:200.5)	25.9
SAMP_HUMAN	Serum amyloid P-component OS=Homo sapiens GN=APCS PE=1 SV=2	25.4	6.1	74.1 (M:74.1)	9.0
SAA2_HUMAN	Serum amyloid A-2 protein OS=Homo sapiens GN=SAA2 PE=1 SV=1	13.5	9.7	260.3 (M:260.3)	36.1
SAA1_HUMAN	Serum amyloid A-1 protein OS=Homo sapiens GN=SAA1 PE=1 SV=1	13.5	6.4	340.3 (M:340.3)	53.3
S12A1_HUMAN	Solute carrier family 12 member 1 OS=Homo sapiens GN=SLC12A1 PE=1 SV=2	121.4	7.8	134.6 (M:134.6)	4.1
S10AB_HUMAN	Protein S100-A11 OS=Homo sapiens GN=S100A11 PE=1 SV=2	11.7	7.5	192.8 (M:192.8)	15.2
S10AA_HUMAN	Protein S100-A10 OS=Homo sapiens GN=S100A10 PE=1 SV=2	11.2	7.7	42.0 (M:42.0)	17.5
S10A9_HUMAN	Protein S100-A9 OS=Homo sapiens GN=S100A9 PE=1 SV=1	13.2	5.7	398.9 (M:398.9)	24.6
S10A8_HUMAN	Protein S100-A8 OS=Homo sapiens GN=S100A8 PE=1 SV=1	10.8	6.6	125.7 (M:125.7)	31.2
S10A6_HUMAN	Protein S100-A6 OS=Homo sapiens GN=S100A6 PE=1 SV=1	10.2	5.2	45.2 (M:45.2)	7.8
RS27A_HUMAN	Ubiquitin-40S ribosomal protein S27a OS=Homo sapiens GN=RPS27A PE=1 SV=2	18.0	10.4	173.1 (M:173.1)	16.0
RS18_HUMAN	40S ribosomal protein S18 OS=Homo sapiens GN=RPS18 PE=1 SV=3	17.7	11.5	51.8 (M:51.8)	10.5
RHOA_HUMAN	Transforming protein RhoA OS=Homo sapiens GN=RHOA PE=1 SV=1	21.8	5.8	129.4 (M:129.4)	14.0
RGPA2_HUMAN	Ral GTPase-activating protein subunit alpha-2 OS=Homo sapiens GN=RALGAPA2 PE=1 SV=2	210.6	5.7	28.5 (M:28.5)	0.4
RETN_HUMAN	Resistin OS=Homo sapiens GN=RETN PE=2 SV=1	11.4	7.7	82.7 (M:82.7)	10.2
RET5_HUMAN	Retinol-binding protein 5 OS=Homo sapiens GN=RBP5 PE=1 SV=3	15.9	6.1	70.1 (M:70.1)	6.7
RET4_HUMAN	Retinol-binding protein 4 OS=Homo sapiens GN=RBP4 PE=1 SV=3	23.0	5.7	1174.1 (M:1174.1)	29.4
RASL2_HUMAN	Ras GTPase-activating protein 4 OS=Homo sapiens GN=RASA4 PE=2 SV=2	90.4	9.0	27.4 (M:27.4)	0.9
RAP1A_HUMAN	Ras-related protein Rap-1A OS=Homo sapiens GN=RAP1A PE=1 SV=1	21.0	7.2	146.6 (M:146.6)	13.6
RADI_HUMAN	Radixin OS=Homo sapiens GN=RDX PE=1 SV=1	68.5	6.0	681.4 (M:681.4)	17.5
RAC1_HUMAN	Ras-related C3 botulinum toxin substrate 1 OS=Homo sapiens	21.4	9.7	126.3	32.8

	GN=RAC1 PE=1 SV=1			(M:126.3)	
RAB10_HUMAN	Ras-related protein Rab-10 OS=Homo sapiens GN=RAB10 PE=1 SV=1	22.5	9.4	58.3 (M:58.3)	5.5
QSOX1_HUMAN	Sulfhydryl oxidase 1 OS=Homo sapiens GN=QSOX1 PE=1 SV=3	82.5	9.9	31.0 (M:31.0)	1.1
QOR_HUMAN	Quinone oxidoreductase OS=Homo sapiens GN=CRYZ PE=1 SV=1	35.2	9.2	47.7 (M:47.7)	4.3
PTGDS_HUMAN	Prostaglandin-H2 D-isomerase OS=Homo sapiens GN=PTGDS PE=1 SV=1	21.0	8.9	217.9 (M:217.9)	17.4
PSCA_HUMAN	Prostate stem cell antigen OS=Homo sapiens GN=PSCA PE=1 SV=1	12.9	4.9	80.2 (M:80.2)	14.6
PRTN3_HUMAN	Myeloblastin OS=Homo sapiens GN=PRTN3 PE=1 SV=3	27.8	10.3	36.3 (M:36.3)	4.7
PROM1_HUMAN	Prominin-1 OS=Homo sapiens GN=PROM1 PE=1 SV=1	97.1	7.2	261.0 (M:261.0)	7.9
PROF1_HUMAN	Profilin-1 OS=Homo sapiens GN=PFN1 PE=1 SV=2	15.0	9.4	121.6 (M:121.6)	30.0
PRDX6_HUMAN	Peroxiredoxin-6 OS=Homo sapiens GN=PRDX6 PE=1 SV=3	25.0	6.0	28.8 (M:28.8)	5.4
PRDX5_HUMAN	Peroxiredoxin-5, mitochondrial OS=Homo sapiens GN=PRDX5 PE=1 SV=4	22.1	9.9	51.1 (M:51.1)	15.9
PRDX3_HUMAN	Thioredoxin-dependent peroxide reductase, mitochondrial OS=Homo sapiens GN=PRDX3 PE=1 SV=3	27.7	8.9	59.8 (M:59.8)	9.0
PRDX1_HUMAN	Peroxiredoxin-1 OS=Homo sapiens GN=PRDX1 PE=1 SV=1	22.1	9.2	114.4 (M:114.4)	26.1
PPIB_HUMAN	Peptidyl-prolyl cis-trans isomerase B OS=Homo sapiens GN=PPIB PE=1 SV=2	23.7	9.9	154.4 (M:154.4)	23.1
PPIA_HUMAN	Peptidyl-prolyl cis-trans isomerase A OS=Homo sapiens GN=PPIA PE=1 SV=2	18.0	9.0	462.0 (M:462.0)	40.0
PODXL_HUMAN	Podocalyxin OS=Homo sapiens GN=PODXL PE=1 SV=2	58.6	5.2	111.9 (M:111.9)	5.2
PLSL_HUMAN	Plastin-2 OS=Homo sapiens GN=LCP1 PE=1 SV=6	70.2	5.2	49.3 (M:49.3)	4.0
PLMN_HUMAN	Plasminogen OS=Homo sapiens GN=PLG PE=1 SV=2	90.5	7.3	88.8 (M:88.8)	6.5
PIP_HUMAN	Prolactin-inducible protein OS=Homo sapiens GN=PIP PE=1 SV=1	16.6	9.3	1028.2 (M:1028.2)	44.5
PIGR_HUMAN	Polymeric immunoglobulin receptor OS=Homo sapiens GN=PIGR PE=1 SV=4	83.2	5.5	1805.5 (M:1805.5)	24.0
PHP14_HUMAN	14 kDa phosphohistidine phosphatase OS=Homo sapiens GN=PHPT1 PE=1 SV=1	13.8	5.6	116.4 (M:116.4)	25.6
PGS1_HUMAN	Biglycan OS=Homo sapiens GN=BGN PE=1 SV=2	41.6	7.9	441.2 (M:441.2)	22.6
PGRP1_HUMAN	Peptidoglycan recognition protein 1 OS=Homo sapiens GN=PGLYRP1 PE=1 SV=1	21.7	10.0	159.3 (M:159.3)	7.7
PGK1_HUMAN	Phosphoglycerate kinase 1 OS=Homo sapiens GN=PGK1 PE=1 SV=3	44.6	9.2	309.3 (M:309.3)	17.3

PGBM_HUMAN	Basement membrane-specific heparan sulfate proteoglycan core protein OS=Homo sapiens GN=HSPG2 PE=1 SV=4	468.5	6.1	200.9 (M:200.9)	1.0
PGAM1_HUMAN	Phosphoglycerate mutase 1 OS=Homo sapiens GN=PGAM1 PE=1 SV=2	28.8	6.8	139.4 (M:139.4)	13.8
PERM_HUMAN	Myeloperoxidase OS=Homo sapiens GN=MPO PE=1 SV=1	83.8	10.1	46.2 (M:46.2)	2.7
PEPC_HUMAN	Gastricsin OS=Homo sapiens GN=PGC PE=1 SV=1	42.4	4.2	123.0 (M:123.0)	4.4
PEDF_HUMAN	Pigment epithelium-derived factor OS=Homo sapiens GN=SERPINF1 PE=1 SV=4	46.3	6.0	285.1 (M:285.1)	8.4
PEBP1_HUMAN	Phosphatidylethanolamine-binding protein 1 OS=Homo sapiens GN=PEBP1 PE=1 SV=3	21.0	7.8	111.8 (M:111.8)	9.6
PDZ1P_HUMAN	Putative PDZ domain-containing protein 1P OS=Homo sapiens GN=PDZK1P1 PE=5 SV=2	44.0	5.3	76.7 (M:76.7)	8.2
PDIA3_HUMAN	Protein disulfide-isomerase A3 OS=Homo sapiens GN=PDIA3 PE=1 SV=4	56.7	5.9	39.7 (M:39.7)	2.2
PDCD6_HUMAN	Programmed cell death protein 6 OS=Homo sapiens GN=PDCD6 PE=1 SV=1	21.9	5.0	180.6 (M:180.6)	31.9
PDC6I_HUMAN	Programmed cell death 6-interacting protein OS=Homo sapiens GN=PDCD6IP PE=1 SV=1	96.0	6.1	511.2 (M:511.2)	15.4
PCP_HUMAN	Lysosomal Pro-X carboxypeptidase OS=Homo sapiens GN=PRCP PE=1 SV=1	55.8	6.9	108.9 (M:108.9)	2.4
OSTP_HUMAN	Osteopontin OS=Homo sapiens GN=SPP1 PE=1 SV=1	35.4	4.2	244.6 (M:244.6)	15.0
OR5C1_HUMAN	Olfactory receptor 5C1 OS=Homo sapiens GN=OR5C1 PE=2 SV=1	35.0	9.9	31.8 (M:31.8)	2.2
NID1_HUMAN	Nidogen-1 OS=Homo sapiens GN=NID1 PE=1 SV=3	136.3	5.0	581.6 (M:581.6)	3.4
NHRF1_HUMAN	Na(+)/H(+) exchange regulatory cofactor NHE-RF1 OS=Homo sapiens GN=SLC9A3R1 PE=1 SV=4	38.8	5.5	323.7 (M:323.7)	33.0
NEP_HUMAN	Neprilysin OS=Homo sapiens GN=MME PE=1 SV=2	85.5	5.4	641.8 (M:641.8)	14.4
NAPSA_HUMAN	Napsin-A OS=Homo sapiens GN=NAPSA PE=1 SV=1	45.4	6.2	565.9 (M:565.9)	10.0
MYL6_HUMAN	Myosin light polypeptide 6 OS=Homo sapiens GN=MYL6 PE=1 SV=2	16.9	4.4	172.7 (M:172.7)	26.5
MYH9_HUMAN	Myosin-9 OS=Homo sapiens GN=MYH9 PE=1 SV=4	226.4	5.4	133.1 (M:133.1)	2.4
MYG_HUMAN	Myoglobin OS=Homo sapiens GN=MB PE=1 SV=2	17.2	7.9	33.5 (M:33.5)	9.7
MXRA8_HUMAN	Matrix-remodeling-associated protein 8 OS=Homo sapiens GN=MXRA8 PE=2 SV=1	49.1	6.8	259.6 (M:259.6)	13.6
MUC6_HUMAN	Mucin-6 OS=Homo sapiens GN=MUC6 PE=1 SV=3	256.9	7.5	129.1 (M:129.1)	1.4
MUC1_HUMAN	Mucin-1 OS=Homo sapiens GN=MUC1 PE=1 SV=3	122.0	7.0	405.6 (M:405.6)	5.9
MORC1_HUMA	MORC family CW-type zinc finger protein 1 OS=Homo	112.8	9.0	33.8 (M:33.8)	0.8

N	sapiens GN=MORC1 PE=2 SV=2				
MOES_HUMAN	Moesin OS=Homo sapiens GN=MSN PE=1 SV=3	67.8	6.0	1409.9 (M:1409.9)	34.3
MMP7_HUMAN	Matrilysin OS=Homo sapiens GN=MMP7 PE=1 SV=1	29.7	8.8	63.4 (M:63.4)	9.4
MMP20_HUMAN	Matrix metalloproteinase-20 OS=Homo sapiens GN=MMP20 PE=1 SV=3	54.4	9.4	27.8 (M:27.8)	1.9
MIF_HUMAN	Macrophage migration inhibitory factor OS=Homo sapiens GN=MIF PE=1 SV=4	12.5	9.1	115.7 (M:115.7)	17.4
MGA_HUMAN	Maltase-glucoamylase, intestinal OS=Homo sapiens GN=MGAM PE=1 SV=5	209.7	5.2	111.1 (M:111.1)	2.2
MDHM_HUMAN	Malate dehydrogenase, mitochondrial OS=Homo sapiens GN=MDH2 PE=1 SV=3	35.5	9.8	56.0 (M:56.0)	7.7
MDHC_HUMAN	Malate dehydrogenase, cytoplasmic OS=Homo sapiens GN=MDH1 PE=1 SV=4	36.4	7.7	32.8 (M:32.8)	2.4
MCCB_HUMAN	Methylcrotonoyl-CoA carboxylase beta chain, mitochondrial OS=Homo sapiens GN=MCCC2 PE=1 SV=1	61.3	8.5	26.0 (M:26.0)	1.6
MASP2_HUMAN	Mannan-binding lectin serine protease 2 OS=Homo sapiens GN=MASP2 PE=1 SV=4	75.7	5.3	1872.2 (M:1872.2)	10.1
MAL2_HUMAN	Protein MAL2 OS=Homo sapiens GN=MAL2 PE=1 SV=1	19.1	5.8	35.4 (M:35.4)	6.2
MA1A1_HUMAN	Mannosyl-oligosaccharide 1,2-alpha-mannosidase IA OS=Homo sapiens GN=MAN1A1 PE=1 SV=3	72.9	6.0	187.0 (M:187.0)	12.1
LYSC_HUMAN	Lysozyme C OS=Homo sapiens GN=LYZ PE=1 SV=1	16.5	10.6	157.8 (M:157.8)	16.2
LMAN2_HUMAN	Vesicular integral-membrane protein VIP36 OS=Homo sapiens GN=LMAN2 PE=1 SV=1	40.2	6.5	960.4 (M:960.4)	46.3
LG3BP_HUMAN	Galectin-3-binding protein OS=Homo sapiens GN=LGALS3BP PE=1 SV=1	65.3	5.0	362.9 (M:362.9)	14.4
LEG3_HUMAN	Galectin-3 OS=Homo sapiens GN=LGALS3 PE=1 SV=5	26.1	9.1	90.6 (M:90.6)	8.4
LDHB_HUMAN	L-lactate dehydrogenase B chain OS=Homo sapiens GN=LDHB PE=1 SV=2	36.6	5.7	356.2 (M:356.2)	21.9
LBP_HUMAN	Lipopolysaccharide-binding protein OS=Homo sapiens GN=LBP PE=1 SV=3	53.3	6.2	89.5 (M:89.5)	5.6
LAMP1_HUMAN	Lysosome-associated membrane glycoprotein 1 OS=Homo sapiens GN=LAMP1 PE=1 SV=3	44.9	9.9	69.6 (M:69.6)	2.6
LAC2_HUMAN	Ig lambda-2 chain C regions OS=Homo sapiens GN=IGLC2 PE=1 SV=1	11.3	7.7	1415.9 (M:1415.9)	74.5
KV402_HUMAN	Ig kappa chain V-IV region Len OS=Homo sapiens PE=1 SV=2	12.6	9.0	267.3 (M:267.3)	15.8
KV309_HUMAN	Ig kappa chain V-III region VG (Fragment) OS=Homo sapiens PE=1 SV=1	12.6	4.7	37.2 (M:37.2)	7.8
KV302_HUMAN	Ig kappa chain V-III region SIE OS=Homo sapiens PE=1 SV=1	11.8	9.5	345.3 (M:345.3)	39.4
KV204_HUMAN	Ig kappa chain V-II region TEW OS=Homo sapiens PE=1 SV=1	12.3	5.6	127.3 (M:127.3)	32.7
KV113_HUMAN	Ig kappa chain V-I region Lay OS=Homo sapiens PE=1 SV=1	11.8	9.2	64.3 (M:64.3)	8.3

KV106_HUMAN	Ig kappa chain V-I region EU OS=Homo sapiens PE=1 SV=1	11.8	9.2	149.7 (M:149.7)	26.9
KV101_HUMAN	Ig kappa chain V-I region AG OS=Homo sapiens PE=1 SV=1	12.0	5.6	114.0 (M:114.0)	16.7
KPYM_HUMAN	Pyruvate kinase isozymes M1/M2 OS=Homo sapiens GN=PKM PE=1 SV=4	57.9	9.0	62.5 (M:62.5)	5.8
KNG1_HUMAN	Kininogen-1 OS=Homo sapiens GN=KNG1 PE=1 SV=2	71.9	6.4	153.2 (M:153.2)	9.0
KCRB_HUMAN	Creatine kinase B-type OS=Homo sapiens GN=CKB PE=1 SV=1	42.6	5.2	69.2 (M:69.2)	4.7
K2C8_HUMAN	Keratin, type II cytoskeletal 8 OS=Homo sapiens GN=KRT8 PE=1 SV=7	53.7	5.4	813.3 (M:813.3)	31.3
K2C7_HUMAN	Keratin, type II cytoskeletal 7 OS=Homo sapiens GN=KRT7 PE=1 SV=5	51.4	5.3	159.2 (M:159.2)	11.9
K2C79_HUMAN	Keratin, type II cytoskeletal 79 OS=Homo sapiens GN=KRT79 PE=1 SV=2	57.8	6.9	88.2 (M:88.2)	4.1
K2C4_HUMAN	Keratin, type II cytoskeletal 4 OS=Homo sapiens GN=KRT4 PE=1 SV=4	57.2	6.2	61.3 (M:61.3)	2.2
K2C1_HUMAN	Keratin, type II cytoskeletal 1 OS=Homo sapiens GN=KRT1 PE=1 SV=6	66.0	8.8	76.9 (M:76.9)	3.7
K1C19_HUMAN	Keratin, type I cytoskeletal 19 OS=Homo sapiens GN=KRT19 PE=1 SV=4	44.1	4.9	622.6 (M:622.6)	48.0
K1C18_HUMAN	Keratin, type I cytoskeletal 18 OS=Homo sapiens GN=KRT18 PE=1 SV=2	48.0	5.2	307.0 (M:307.0)	16.5
K1C13_HUMAN	Keratin, type I cytoskeletal 13 OS=Homo sapiens GN=KRT13 PE=1 SV=4	49.6	4.8	143.5 (M:143.5)	11.1
K1C10_HUMAN	Keratin, type I cytoskeletal 10 OS=Homo sapiens GN=KRT10 PE=1 SV=6	58.8	5.0	60.7 (M:60.7)	4.1
ITIH4_HUMAN	Inter-alpha-trypsin inhibitor heavy chain H4 OS=Homo sapiens GN=ITIH4 PE=1 SV=4	103.3	6.5	149.0 (M:149.0)	5.6
IST1_HUMAN	IST1 homolog OS=Homo sapiens GN=IST1 PE=1 SV=1	39.7	5.1	608.2 (M:608.2)	19.5
IPSP_HUMAN	Plasma serine protease inhibitor OS=Homo sapiens GN=SERPINA5 PE=1 SV=3	45.6	9.8	125.5 (M:125.5)	12.3
IGLL5_HUMAN	Immunoglobulin lambda-like polypeptide 5 OS=Homo sapiens GN=IGLL5 PE=2 SV=2	23.0	10.0	1200.6 (M:1200.6)	27.1
IGKC_HUMAN	Ig kappa chain C region OS=Homo sapiens GN=IGKC PE=1 SV=1	11.6	5.5	3056.5 (M:3056.5)	64.2
IGJ_HUMAN	Immunoglobulin J chain OS=Homo sapiens GN=IGJ PE=1 SV=4	18.1	5.0	479.3 (M:479.3)	28.3
IGHM_HUMAN	Ig mu chain C region OS=Homo sapiens GN=IGHM PE=1 SV=3	49.3	6.4	193.4 (M:193.4)	17.3
IGHG4_HUMAN	Ig gamma-4 chain C region OS=Homo sapiens GN=IGHG4 PE=1 SV=1	35.9	7.8	890.5 (M:890.5)	22.6
IGHG1_HUMA	Ig gamma-1 chain C region OS=Homo sapiens GN=IGHG1	36.1	9.4	1283.8	45.2

N	PE=1 SV=1			(M:1283.8)	
IGHA1_HUMAN	Ig alpha-1 chain C region OS=Homo sapiens GN=IGHA1 PE=1 SV=2	37.6	6.1	4619.3 (M:4619.3)	52.4
IDHC_HUMAN	Isocitrate dehydrogenase [NADP] cytoplasmic OS=Homo sapiens GN=IDH1 PE=1 SV=2	46.6	6.6	30.5 (M:30.5)	2.2
IC1_HUMAN	Plasma protease C1 inhibitor OS=Homo sapiens GN=SERPING1 PE=1 SV=2	55.1	6.1	277.0 (M:277.0)	10.8
HSPB1_HUMAN	Heat shock protein beta-1 OS=Homo sapiens GN=HSPB1 PE=1 SV=2	22.8	6.0	294.4 (M:294.4)	30.2
HSP7C_HUMAN	Heat shock cognate 71 kDa protein OS=Homo sapiens GN=HSPA8 PE=1 SV=1	70.9	5.2	357.6 (M:357.6)	16.7
HSP71_HUMAN	Heat shock 70 kDa protein 1A/1B OS=Homo sapiens GN=HSPA1A PE=1 SV=5	70.0	5.4	197.3 (M:197.3)	12.6
HS90B_HUMAN	Heat shock protein HSP 90-beta OS=Homo sapiens GN=HSP90AB1 PE=1 SV=4	83.2	4.8	51.9 (M:51.9)	5.0
HS90A_HUMAN	Heat shock protein HSP 90-alpha OS=Homo sapiens GN=HSP90AA1 PE=1 SV=5	84.6	4.8	52.2 (M:52.2)	4.0
HPT_HUMAN	Haptoglobin OS=Homo sapiens GN=HP PE=1 SV=1	45.2	6.1	71.3 (M:71.3)	9.9
HEXB_HUMAN	Beta-hexosaminidase subunit beta OS=Homo sapiens GN=HEXB PE=1 SV=3	63.1	6.3	33.8 (M:33.8)	2.0
HEMO_HUMAN	Hemopexin OS=Homo sapiens GN=HPX PE=1 SV=2	51.6	6.6	91.6 (M:91.6)	4.3
HBD_HUMAN	Hemoglobin subunit delta OS=Homo sapiens GN=HBD PE=1 SV=2	16.0	9.1	1433.7 (M:1433.7)	42.9
HBB_HUMAN	Hemoglobin subunit beta OS=Homo sapiens GN=HBB PE=1 SV=2	16.0	6.9	2203.7 (M:2203.7)	89.8
HBA_HUMAN	Hemoglobin subunit alpha OS=Homo sapiens GN=HBA1 PE=1 SV=2	15.2	9.4	1254.4 (M:1254.4)	48.6
H4_HUMAN	Histone H4 OS=Homo sapiens GN=HIST1H4A PE=1 SV=2	11.4	11.8	426.7 (M:426.7)	35.0
H33_HUMAN	Histone H3.3 OS=Homo sapiens GN=H3F3A PE=1 SV=2	15.3	11.9	402.7 (M:402.7)	30.1
H2B1C_HUMAN	Histone H2B type 1-C/E/F/G/I OS=Homo sapiens GN=HIST1H2BC PE=1 SV=4	13.9	10.8	694.5 (M:694.5)	32.5
H2B1B_HUMAN	Histone H2B type 1-B OS=Homo sapiens GN=HIST1H2BB PE=1 SV=2	13.9	10.8	703.8 (M:703.8)	32.5
H2AW_HUMAN	Core histone macro-H2A.2 OS=Homo sapiens GN=H2AFY2 PE=1 SV=3	40.0	10.3	77.1 (M:77.1)	2.4
H2AV_HUMAN	Histone H2A.V OS=Homo sapiens GN=H2AFV PE=1 SV=3	13.5	11.0	154.2 (M:154.2)	23.4
H2A1D_HUMAN	Histone H2A type 1-D OS=Homo sapiens GN=HIST1H2AD PE=1 SV=2	14.1	11.4	906.3 (M:906.3)	35.4
H2A1B_HUMAN	Histone H2A type 1-B/E OS=Homo sapiens GN=HIST1H2AB PE=1 SV=2	14.1	11.5	888.0 (M:888.0)	35.4
H2A1A_HUMA	Histone H2A type 1-A OS=Homo sapiens GN=HIST1H2AA	14.2	11.3	234.6	29.8

N	PE=1 SV=3			(M:234.6)	
H12_HUMAN	Histone H1.2 OS=Homo sapiens GN=HIST1H1C PE=1 SV=2	21.4	11.4	215.2 (M:215.2)	17.8
GSTP1_HUMAN	Glutathione S-transferase P OS=Homo sapiens GN=GSTP1 PE=1 SV=2	23.3	5.3	189.3 (M:189.3)	19.5
GSTA2_HUMAN	Glutathione S-transferase A2 OS=Homo sapiens GN=GSTA2 PE=1 SV=4	25.6	9.1	89.5 (M:89.5)	11.7
GRAN_HUMAN	Grancalcin OS=Homo sapiens GN=GCA PE=1 SV=2	24.0	4.9	43.1 (M:43.1)	4.1
GPC5C_HUMAN	G-protein coupled receptor family C group 5 member C OS=Homo sapiens GN=GPRC5C PE=1 SV=2	48.2	9.6	440.0 (M:440.0)	23.4
GPC5B_HUMAN	G-protein coupled receptor family C group 5 member B OS=Homo sapiens GN=GPRC5B PE=2 SV=2	44.8	9.6	220.0 (M:220.0)	8.7
GPC4_HUMAN	Glypican-4 OS=Homo sapiens GN=GPC4 PE=1 SV=4	62.4	6.3	62.7 (M:62.7)	3.1
GPC3_HUMAN	Glypican-3 OS=Homo sapiens GN=GPC3 PE=1 SV=1	65.5	5.9	147.4 (M:147.4)	7.8
GPC1_HUMAN	Glypican-1 OS=Homo sapiens GN=GPC1 PE=1 SV=2	61.6	7.8	45.2 (M:45.2)	5.4
GNAL_HUMAN	Guanine nucleotide-binding protein G(olf) subunit alpha OS=Homo sapiens GN=GNAL PE=1 SV=1	44.3	6.2	67.4 (M:67.4)	2.9
GILT_HUMAN	Gamma-interferon-inducible lysosomal thiol reductase OS=Homo sapiens GN=IFI30 PE=1 SV=3	27.9	4.6	29.3 (M:29.3)	4.4
GGT1_HUMAN	Gamma-glutamyltranspeptidase 1 OS=Homo sapiens GN=GGT1 PE=1 SV=2	61.4	6.7	314.0 (M:314.0)	10.0
GELS_HUMAN	Gelsolin OS=Homo sapiens GN=GSN PE=1 SV=1	85.6	5.9	605.1 (M:605.1)	11.4
GDPD3_HUMAN	Glycerophosphodiester phosphodiesterase domain-containing protein 3 OS=Homo sapiens GN=GDPD3 PE=2 SV=3	36.6	9.2	90.0 (M:90.0)	4.4
GDIA_HUMAN	Rab GDP dissociation inhibitor alpha OS=Homo sapiens GN=GDI1 PE=1 SV=2	50.6	4.9	124.2 (M:124.2)	4.3
GBG12_HUMAN	Guanine nucleotide-binding protein G(I)/G(S)/G(O) subunit gamma-12 OS=Homo sapiens GN=GNG12 PE=1 SV=3	8.0	10.1	53.4 (M:53.4)	22.2
G6PI_HUMAN	Glucose-6-phosphate isomerase OS=Homo sapiens GN=GPI PE=1 SV=4	63.1	9.1	143.0 (M:143.0)	5.7
G3P_HUMAN	Glyceraldehyde-3-phosphate dehydrogenase OS=Homo sapiens GN=GAPDH PE=1 SV=3	36.0	9.3	1189.8 (M:1189.8)	41.2
FINC_HUMAN	Fibronectin OS=Homo sapiens GN=FN1 PE=1 SV=4	262.5	5.4	4823.4 (M:4823.4)	26.7
FIBG_HUMAN	Fibrinogen gamma chain OS=Homo sapiens GN=FGG PE=1 SV=3	51.5	5.3	2452.2 (M:2452.2)	54.1
FIBB_HUMAN	Fibrinogen beta chain OS=Homo sapiens GN=FGB PE=1 SV=2	55.9	9.3	3939.1 (M:3939.1)	57.0
FIBA_HUMAN	Fibrinogen alpha chain OS=Homo sapiens GN=FGA PE=1 SV=2	94.9	5.6	3520.0 (M:3520.0)	39.7
FGL2_HUMAN	Fibroleukin OS=Homo sapiens GN=FGL2 PE=1 SV=1	50.2	7.7	156.8 (M:156.8)	5.7
FETUA_HUMA	Alpha-2-HS-glycoprotein OS=Homo sapiens GN=AHSG PE=1	39.3	5.4	502.3	19.9

N	SV=1			(M:502.3)	
FCN2_HUMAN	Ficolin-2 OS=Homo sapiens GN=FCN2 PE=1 SV=2	34.0	6.4	58.5 (M:58.5)	4.2
FBLN5_HUMAN	Fibulin-5 OS=Homo sapiens GN=FBLN5 PE=1 SV=1	50.1	4.4	29.9 (M:29.9)	2.0
FBLN3_HUMAN	EGF-containing fibulin-like extracellular matrix protein 1 OS=Homo sapiens GN=EFEMP1 PE=1 SV=2	54.6	4.8	160.8 (M:160.8)	12.6
FABPH_HUMAN	Fatty acid-binding protein, heart OS=Homo sapiens GN=FABP3 PE=1 SV=4	14.8	6.4	41.7 (M:41.7)	6.8
F16P1_HUMAN	Fructose-1,6-bisphosphatase 1 OS=Homo sapiens GN=FBP1 PE=1 SV=5	36.8	6.7	182.9 (M:182.9)	9.8
EZRI_HUMAN	Ezrin OS=Homo sapiens GN=EZR PE=1 SV=4	69.4	5.9	1750.2 (M:1750.2)	37.0
ESPB1_HUMAN	Epididymal sperm-binding protein 1 OS=Homo sapiens GN=ELSPBP1 PE=1 SV=2	26.1	6.2	34.6 (M:34.6)	3.6
ES8L2_HUMAN	Epidermal growth factor receptor kinase substrate 8-like protein 2 OS=Homo sapiens GN=EPS8L2 PE=1 SV=2	80.6	6.4	138.8 (M:138.8)	8.3
ENPL_HUMAN	Endoplasmin OS=Homo sapiens GN=HSP90B1 PE=1 SV=1	92.4	4.6	71.5 (M:71.5)	1.2
ENOA_HUMAN	Alpha-enolase OS=Homo sapiens GN=ENO1 PE=1 SV=2	47.1	7.7	1047.2 (M:1047.2)	35.3
ELNE_HUMAN	Neutrophil elastase OS=Homo sapiens GN=ELANE PE=1 SV=1	28.5	12.2	68.0 (M:68.0)	6.4
EHD4_HUMAN	EH domain-containing protein 4 OS=Homo sapiens GN=EHD4 PE=1 SV=1	61.1	6.3	300.2 (M:300.2)	13.7
EHD1_HUMAN	EH domain-containing protein 1 OS=Homo sapiens GN=EHD1 PE=1 SV=2	60.6	6.4	83.0 (M:83.0)	12.0
EGF_HUMAN	Pro-epidermal growth factor OS=Homo sapiens GN=EGF PE=1 SV=2	133.9	5.5	117.7 (M:117.7)	2.6
EF2_HUMAN	Elongation factor 2 OS=Homo sapiens GN=EEF2 PE=1 SV=4	95.3	6.4	61.8 (M:61.8)	1.5
EF1A1_HUMAN	Elongation factor 1-alpha 1 OS=Homo sapiens GN=EEF1A1 PE=1 SV=1	50.1	9.7	197.8 (M:197.8)	9.7
ECM1_HUMAN	Extracellular matrix protein 1 OS=Homo sapiens GN=ECM1 PE=1 SV=2	60.6	6.3	117.4 (M:117.4)	8.7
ECHM_HUMAN	Enoyl-CoA hydratase, mitochondrial OS=Homo sapiens GN=ECHS1 PE=1 SV=4	31.4	9.4	77.3 (M:77.3)	5.9
ECHB_HUMAN	Trifunctional enzyme subunit beta, mitochondrial OS=Homo sapiens GN=HADHB PE=1 SV=3	51.3	10.0	83.1 (M:83.1)	1.9
DPP4_HUMAN	Dipeptidyl peptidase 4 OS=Homo sapiens GN=DPP4 PE=1 SV=2	88.2	5.6	43.7 (M:43.7)	1.3
DPEP1_HUMAN	Dipeptidase 1 OS=Homo sapiens GN=DPEP1 PE=1 SV=3	45.6	5.7	350.2 (M:350.2)	13.4
DEFB1_HUMAN	Beta-defensin 1 OS=Homo sapiens GN=DEFB1 PE=1 SV=1	7.4	10.3	69.5 (M:69.5)	33.8
DEF1_HUMAN	Neutrophil defensin 1 OS=Homo sapiens GN=DEFA1 PE=1 SV=1	10.2	7.6	86.6 (M:86.6)	20.2
DDC_HUMAN	Aromatic-L-amino-acid decarboxylase OS=Homo sapiens	53.9	6.9	34.0 (M:34.0)	1.7

	GN=DDC PE=1 SV=2				
CYTC_HUMAN	Cystatin-C OS=Homo sapiens GN=CST3 PE=1 SV=1	15.8	9.9	135.4 (M:135.4)	30.8
CYTB_HUMAN	Cystatin-B OS=Homo sapiens GN=CSTB PE=1 SV=2	11.1	7.9	62.0 (M:62.0)	12.2
CUBN_HUMAN	Cubilin OS=Homo sapiens GN=CUBN PE=1 SV=5	398.5	5.0	534.4 (M:534.4)	4.7
CRYAB_HUMAN	Alpha-crystallin B chain OS=Homo sapiens GN=CRYAB PE=1 SV=2	20.1	6.9	209.1 (M:209.1)	24.6
CRIS1_HUMAN	Cysteine-rich secretory protein 1 OS=Homo sapiens GN=CRISP1 PE=1 SV=1	28.5	5.5	453.8 (M:453.8)	23.7
CRAC1_HUMAN	Cartilage acidic protein 1 OS=Homo sapiens GN=CRTAC1 PE=1 SV=2	71.4	4.8	61.6 (M:61.6)	2.9
CPVL_HUMAN	Probable serine carboxypeptidase CPVL OS=Homo sapiens GN=CPVL PE=1 SV=2	54.1	5.3	133.0 (M:133.0)	6.7
CPNE3_HUMAN	Copine-3 OS=Homo sapiens GN=CPNE3 PE=1 SV=1	60.1	5.5	144.7 (M:144.7)	7.6
COX5B_HUMAN	Cytochrome c oxidase subunit 5B, mitochondrial OS=Homo sapiens GN=COX5B PE=1 SV=2	13.7	10.2	53.9 (M:53.9)	9.3
COX5A_HUMAN	Cytochrome c oxidase subunit 5A, mitochondrial OS=Homo sapiens GN=COX5A PE=1 SV=2	16.8	6.4	56.3 (M:56.3)	6.0
COIA1_HUMAN	Collagen alpha-1(XVIII) chain OS=Homo sapiens GN=COL18A1 PE=1 SV=5	178.1	5.6	210.5 (M:210.5)	3.9
COF1_HUMAN	Cofilin-1 OS=Homo sapiens GN=CFL1 PE=1 SV=3	18.5	9.1	112.9 (M:112.9)	18.7
CO9_HUMAN	Complement component C9 OS=Homo sapiens GN=C9 PE=1 SV=2	63.1	5.3	408.8 (M:408.8)	23.4
CO4B_HUMAN	Complement C4-B OS=Homo sapiens GN=C4B PE=1 SV=2	192.6	7.0	1589.2 (M:1589.2)	19.4
CO4A_HUMAN	Complement C4-A OS=Homo sapiens GN=C4A PE=1 SV=2	192.7	6.7	1560.9 (M:1560.9)	18.5
CO3_HUMAN	Complement C3 OS=Homo sapiens GN=C3 PE=1 SV=2	187.0	6.0	185.5 (M:185.5)	3.2
CNDP2_HUMAN	Cytosolic non-specific dipeptidase OS=Homo sapiens GN=CNDP2 PE=1 SV=2	52.8	5.6	153.9 (M:153.9)	15.8
CN37_HUMAN	2',3'-cyclic-nucleotide 3'-phosphodiesterase OS=Homo sapiens GN=CNP PE=1 SV=2	47.5	9.8	203.9 (M:203.9)	7.6
CLUS_HUMAN	Clusterin OS=Homo sapiens GN=CLU PE=1 SV=1	52.5	5.9	2396.7 (M:2396.7)	34.3
CLIC4_HUMAN	Chloride intracellular channel protein 4 OS=Homo sapiens GN=CLIC4 PE=1 SV=4	28.8	5.3	130.9 (M:130.9)	8.7
CLIC1_HUMAN	Chloride intracellular channel protein 1 OS=Homo sapiens GN=CLIC1 PE=1 SV=4	26.9	4.9	131.7 (M:131.7)	12.4
CHMP5_HUMAN	Charged multivesicular body protein 5 OS=Homo sapiens GN=CHMP5 PE=1 SV=1	24.6	4.5	206.9 (M:206.9)	15.5
CHM4B_HUMA	Charged multivesicular body protein 4b OS=Homo sapiens	24.9	4.6	84.5 (M:84.5)	10.7

N	GN=CHMP4B PE=1 SV=1				
CHM2A_HUMAN	Charged multivesicular body protein 2a OS=Homo sapiens GN=CHMP2A PE=1 SV=1	25.1	5.7	57.7 (M:57.7)	7.2
CHM1B_HUMAN	Charged multivesicular body protein 1b OS=Homo sapiens GN=CHMP1B PE=1 SV=1	22.1	9.0	88.0 (M:88.0)	5.0
CH60_HUMAN	60 kDa heat shock protein, mitochondrial OS=Homo sapiens GN=HSPD1 PE=1 SV=2	61.0	5.6	176.4 (M:176.4)	5.8
CFAB_HUMAN	Complement factor B OS=Homo sapiens GN=CFB PE=1 SV=2	85.5	6.7	278.0 (M:278.0)	13.5
CETP_HUMAN	Cholesteryl ester transfer protein OS=Homo sapiens GN=CETP PE=1 SV=2	54.7	5.7	72.7 (M:72.7)	3.0
CERU_HUMAN	Ceruloplasmin OS=Homo sapiens GN=CP PE=1 SV=1	122.1	5.4	168.7 (M:168.7)	3.9
CDC42_HUMAN	Cell division control protein 42 homolog OS=Homo sapiens GN=CDC42 PE=1 SV=2	21.2	6.2	222.3 (M:222.3)	19.9
CD9_HUMAN	CD9 antigen OS=Homo sapiens GN=CD9 PE=1 SV=4	25.4	7.7	262.7 (M:262.7)	15.4
CD59_HUMAN	CD59 glycoprotein OS=Homo sapiens GN=CD59 PE=1 SV=1	14.2	6.1	121.1 (M:121.1)	19.5
CD14_HUMAN	Monocyte differentiation antigen CD14 OS=Homo sapiens GN=CD14 PE=1 SV=2	40.1	5.8	735.6 (M:735.6)	26.4
CBR1_HUMAN	Carbonyl reductase [NADPH] 1 OS=Homo sapiens GN=CBR1 PE=1 SV=3	30.4	9.5	75.3 (M:75.3)	4.0
CBPZ_HUMAN	Carboxypeptidase Z OS=Homo sapiens GN=CPZ PE=1 SV=2	73.6	9.2	48.6 (M:48.6)	1.5
CATZ_HUMAN	Cathepsin Z OS=Homo sapiens GN=CTSZ PE=1 SV=1	33.8	6.9	97.0 (M:97.0)	7.3
CATG_HUMAN	Cathepsin G OS=Homo sapiens GN=CTSG PE=1 SV=2	28.8	12.1	121.6 (M:121.6)	10.6
CATD_HUMAN	Cathepsin D OS=Homo sapiens GN=CTSD PE=1 SV=1	44.5	6.1	415.4 (M:415.4)	18.2
CATC_HUMAN	Dipeptidyl peptidase 1 OS=Homo sapiens GN=CTSC PE=1 SV=2	51.8	6.6	53.8 (M:53.8)	2.6
CAP1_HUMAN	Adenylyl cyclase-associated protein 1 OS=Homo sapiens GN=CAP1 PE=1 SV=5	51.9	9.1	79.9 (M:79.9)	4.8
CAMP_HUMAN	Cathelicidin antimicrobial peptide OS=Homo sapiens GN=CAMP PE=1 SV=1	19.3	10.3	186.2 (M:186.2)	11.8
CALX_HUMAN	Calnexin OS=Homo sapiens GN=CANX PE=1 SV=2	67.5	4.3	86.0 (M:86.0)	3.7
CALR_HUMAN	Calreticulin OS=Homo sapiens GN=CALR PE=1 SV=1	48.1	4.1	91.2 (M:91.2)	14.9
CALD1_HUMAN	Caldesmon OS=Homo sapiens GN=CALD1 PE=1 SV=3	93.2	5.5	32.3 (M:32.3)	1.0
CALB1_HUMAN	Calbindin OS=Homo sapiens GN=CALB1 PE=1 SV=2	30.0	4.6	86.2 (M:86.2)	5.7
CAH2_HUMAN	Carbonic anhydrase 2 OS=Homo sapiens GN=CA2 PE=1 SV=2	29.2	7.0	278.7 (M:278.7)	13.5
CAH1_HUMAN	Carbonic anhydrase 1 OS=Homo sapiens GN=CA1 PE=1 SV=2	28.9	6.7	32.3 (M:32.3)	3.4

CAB45_HUMAN	45 kDa calcium-binding protein OS=Homo sapiens GN=SDF4 PE=1 SV=1	41.8	4.6	45.4 (M:45.4)	2.8
C1QC_HUMAN	Complement C1q subcomponent subunit C OS=Homo sapiens GN=C1QC PE=1 SV=3	25.8	9.4	39.4 (M:39.4)	3.7
BHMT1_HUMAN	Betaine--homocysteine S-methyltransferase 1 OS=Homo sapiens GN=BHMT PE=1 SV=2	45.0	6.7	353.7 (M:353.7)	19.5
BGAL_HUMAN	Beta-galactosidase OS=Homo sapiens GN=GLB1 PE=1 SV=2	76.0	6.1	27.1 (M:27.1)	1.5
B2MG_HUMAN	Beta-2-microglobulin OS=Homo sapiens GN=B2M PE=1 SV=1	13.7	6.1	896.0 (M:896.0)	44.5
ATPO_HUMAN	ATP synthase subunit O, mitochondrial OS=Homo sapiens GN=ATP5O PE=1 SV=1	23.3	10.5	97.0 (M:97.0)	22.5
ATPK_HUMAN	ATP synthase subunit f, mitochondrial OS=Homo sapiens GN=ATP5J2 PE=2 SV=3	10.9	10.2	58.8 (M:58.8)	11.7
ATPB_HUMAN	ATP synthase subunit beta, mitochondrial OS=Homo sapiens GN=ATP5B PE=1 SV=3	56.5	5.1	163.2 (M:163.2)	13.8
ATPA_HUMAN	ATP synthase subunit alpha, mitochondrial OS=Homo sapiens GN=ATP5A1 PE=1 SV=1	59.7	9.6	121.5 (M:121.5)	6.3
ATP5H_HUMAN	ATP synthase subunit d, mitochondrial OS=Homo sapiens GN=ATP5H PE=1 SV=3	18.5	5.1	128.4 (M:128.4)	18.6
AT1B1_HUMAN	Sodium/potassium-transporting ATPase subunit beta-1 OS=Homo sapiens GN=ATP1B1 PE=1 SV=1	35.0	9.4	109.0 (M:109.0)	11.9
AT1A1_HUMAN	Sodium/potassium-transporting ATPase subunit alpha-1 OS=Homo sapiens GN=ATP1A1 PE=1 SV=1	112.8	5.2	72.2 (M:72.2)	1.9
ASSY_HUMAN	Argininosuccinate synthase OS=Homo sapiens GN=ASS1 PE=1 SV=2	46.5	8.9	57.8 (M:57.8)	4.6
ASAH1_HUMAN	Acid ceramidase OS=Homo sapiens GN=ASAH1 PE=1 SV=5	44.6	8.6	106.2 (M:106.2)	5.3
ARSF_HUMAN	Arylsulfatase F OS=Homo sapiens GN=ARSF PE=1 SV=4	65.9	6.9	26.8 (M:26.8)	4.6
ARPC4_HUMAN	Actin-related protein 2/3 complex subunit 4 OS=Homo sapiens GN=ARPC4 PE=1 SV=3	19.7	9.4	52.0 (M:52.0)	6.5
ARP3_HUMAN	Actin-related protein 3 OS=Homo sapiens GN=ACTR3 PE=1 SV=3	47.3	5.5	67.5 (M:67.5)	6.7
ARF1_HUMAN	ADP-ribosylation factor 1 OS=Homo sapiens GN=ARF1 PE=1 SV=2	20.7	6.4	59.4 (M:59.4)	9.9
AQP2_HUMAN	Aquaporin-2 OS=Homo sapiens GN=AQP2 PE=1 SV=1	28.8	6.5	110.1 (M:110.1)	4.1
APOE_HUMAN	Apolipoprotein E OS=Homo sapiens GN=APOE PE=1 SV=1	36.1	5.5	224.2 (M:224.2)	16.7
APOD_HUMAN	Apolipoprotein D OS=Homo sapiens GN=APOD PE=1 SV=1	21.3	4.9	594.3 (M:594.3)	36.0
APOA4_HUMAN	Apolipoprotein A-IV OS=Homo sapiens GN=APOA4 PE=1 SV=3	45.4	5.2	1086.4 (M:1086.4)	62.1
APOA1_HUMAN	Apolipoprotein A-I OS=Homo sapiens GN=APOA1 PE=1 SV=1	30.8	5.5	345.4 (M:345.4)	28.1
ANXA6_HUMA	Annexin A6 OS=Homo sapiens GN=ANXA6 PE=1 SV=3	75.8	5.3	56.8 (M:56.8)	4.3

N					
ANXA5_HUMAN	Annexin A5 OS=Homo sapiens GN=ANXA5 PE=1 SV=2	35.9	4.8	519.9 (M:519.9)	23.8
ANXA4_HUMAN	Annexin A4 OS=Homo sapiens GN=ANXA4 PE=1 SV=4	35.9	5.8	664.8 (M:664.8)	30.1
ANXA2_HUMAN	Annexin A2 OS=Homo sapiens GN=ANXA2 PE=1 SV=2	38.6	8.5	1103.1 (M:1103.1)	45.4
ANXA1_HUMAN	Annexin A1 OS=Homo sapiens GN=ANXA1 PE=1 SV=2	38.7	6.7	173.5 (M:173.5)	18.5
ANX11_HUMAN	Annexin A11 OS=Homo sapiens GN=ANXA11 PE=1 SV=1	54.4	8.5	448.6 (M:448.6)	24.0
ANT3_HUMAN	Antithrombin-III OS=Homo sapiens GN=SERPINC1 PE=1 SV=1	52.6	6.3	90.1 (M:90.1)	4.1
ANGL7_HUMAN	Angiopoietin-related protein 7 OS=Homo sapiens GN=ANGPTL7 PE=1 SV=1	40.0	8.9	26.9 (M:26.9)	6.4
ANGL6_HUMAN	Angiopoietin-related protein 6 OS=Homo sapiens GN=ANGPTL6 PE=1 SV=1	51.7	9.6	48.5 (M:48.5)	8.7
ANGL2_HUMAN	Angiopoietin-related protein 2 OS=Homo sapiens GN=ANGPTL2 PE=2 SV=1	57.1	7.8	93.1 (M:93.1)	7.1
AMPN_HUMAN	Aminopeptidase N OS=Homo sapiens GN=ANPEP PE=1 SV=4	109.5	5.2	1535.4 (M:1535.4)	23.5
AMPE_HUMAN	Glutamyl aminopeptidase OS=Homo sapiens GN=ENPEP PE=1 SV=3	109.2	5.2	64.1 (M:64.1)	1.3
AMBP_HUMAN	Protein AMBP OS=Homo sapiens GN=AMBP PE=1 SV=1	39.0	5.9	1513.5 (M:1513.5)	43.8
ALDR_HUMAN	Aldose reductase OS=Homo sapiens GN=AKR1B1 PE=1 SV=3	35.8	6.6	154.7 (M:154.7)	12.3
ALDOB_HUMAN	Fructose-bisphosphate aldolase B OS=Homo sapiens GN=ALDOB PE=1 SV=2	39.4	9.0	717.2 (M:717.2)	25.3
ALDOA_HUMAN	Fructose-bisphosphate aldolase A OS=Homo sapiens GN=ALDOA PE=1 SV=2	39.4	9.2	68.6 (M:68.6)	3.0
ALBU_HUMAN	Serum albumin OS=Homo sapiens GN=ALB PE=1 SV=2	69.3	5.9	2531.8 (M:2531.8)	43.0
AL1A1_HUMAN	Retinal dehydrogenase 1 OS=Homo sapiens GN=ALDH1A1 PE=1 SV=2	54.8	6.3	32.6 (M:32.6)	2.0
AK1A1_HUMAN	Alcohol dehydrogenase [NADP(+)] OS=Homo sapiens GN=AKR1A1 PE=1 SV=3	36.5	6.4	225.8 (M:225.8)	16.0
AHNK_HUMAN	Neuroblast differentiation-associated protein AHNAK OS=Homo sapiens GN=AHNAK PE=1 SV=2	628.7	5.7	90.3 (M:90.3)	0.4
AGRIN_HUMAN	Agrin OS=Homo sapiens GN=AGRN PE=1 SV=5	217.1	6.0	305.5 (M:305.5)	4.9
ADT2_HUMAN	ADP/ATP translocase 2 OS=Homo sapiens GN=SLC25A5 PE=1 SV=7	32.8	10.2	59.2 (M:59.2)	5.7
ACY1_HUMAN	Aminoacylase-1 OS=Homo sapiens GN=ACY1 PE=1 SV=1	45.9	5.7	334.0 (M:334.0)	10.5

ACTN4_HUMAN	Alpha-actinin-4 OS=Homo sapiens GN=ACTN4 PE=1 SV=2	104.8	5.2	108.0 (M:108.0)	5.7
ACTC_HUMAN	Actin, alpha cardiac muscle 1 OS=Homo sapiens GN=ACTC1 PE=1 SV=1	42.0	5.1	825.6 (M:825.6)	24.7
ACTB_HUMAN	Actin, cytoplasmic 1 OS=Homo sapiens GN=ACTB PE=1 SV=1	41.7	5.2	1574.9 (M:1574.9)	50.7
ACE_HUMAN	Angiotensin-converting enzyme OS=Homo sapiens GN=ACE PE=1 SV=1	149.6	5.9	36.5 (M:36.5)	1.0
ACE2_HUMAN	Angiotensin-converting enzyme 2 OS=Homo sapiens GN=ACE2 PE=1 SV=2	92.4	5.3	46.0 (M:46.0)	1.5
ACADM_HUMAN	Medium-chain specific acyl-CoA dehydrogenase, mitochondrial OS=Homo sapiens GN=ACADM PE=1 SV=1	46.6	9.4	31.9 (M:31.9)	7.4
A2GL_HUMAN	Leucine-rich alpha-2-glycoprotein OS=Homo sapiens GN=LRG1 PE=1 SV=2	38.2	6.5	509.0 (M:509.0)	23.9
A2AP_HUMAN	Alpha-2-antiplasmin OS=Homo sapiens GN=SERPINF2 PE=1 SV=3	54.5	5.9	35.8 (M:35.8)	2.2
A1BG_HUMAN	Alpha-1B-glycoprotein OS=Homo sapiens GN=A1BG PE=1 SV=4	54.2	5.5	931.6 (M:931.6)	36.0
A1AT_HUMAN	Alpha-1-antitrypsin OS=Homo sapiens GN=SERPINA1 PE=1 SV=3	46.7	5.3	898.7 (M:898.7)	25.8
A1AG1_HUMAN	Alpha-1-acid glycoprotein 1 OS=Homo sapiens GN=ORM1 PE=1 SV=1	23.5	4.8	405.0 (M:405.0)	27.9
6PGL_HUMAN	6-phosphogluconolactonase OS=Homo sapiens GN=PGLS PE=1 SV=2	27.5	5.7	101.8 (M:101.8)	29.8
4F2_HUMAN	4F2 cell-surface antigen heavy chain OS=Homo sapiens GN=SLC3A2 PE=1 SV=3	68.0	4.7	66.1 (M:66.1)	1.9
2B19_HUMAN	HLA class II histocompatibility antigen, DRB1-9 beta chain OS=Homo sapiens GN=HLA-DRB1 PE=1 SV=1	29.8	8.9	68.7 (M:68.7)	7.1
1A01_HUMAN	HLA class I histocompatibility antigen, A-1 alpha chain OS=Homo sapiens GN=HLA-A PE=1 SV=1	40.8	6.1	111.9 (M:111.9)	8.2
1433Z_HUMAN	14-3-3 protein zeta/delta OS=Homo sapiens GN=YWHAZ PE=1 SV=1	27.7	4.6	313.3 (M:313.3)	42.4
1433E_HUMAN	14-3-3 protein epsilon OS=Homo sapiens GN=YWHAE PE=1 SV=1	29.2	4.5	228.3 (M:228.3)	18.8
1433B_HUMAN	14-3-3 protein beta/alpha OS=Homo sapiens GN=YWHAB PE=1 SV=3	28.1	4.6	142.9 (M:142.9)	15.0

Supplemental Table 2. Patient characteristics

	UC	Comparison			*p*
		UTI	Prostate cancer	Normal	
Total, n	129	17	25	20	
Age (yrs)	67.34±11.35	64.41±7.68	63.65±8.85	69.88±9.96	0.178
Gender, Male (%)	85 (65.9%)	7 (41.2%)	25 (100%)	11 (55%)	0.328
Location at presentation, n (%)					
Bladder	70 (54.3%)	-	-	-	-
Ureter or Renal pelvis	59 (45.7%)	-	-	-	-
Pathologic stage, n (%)					
pTa/Tis/T1	63 (48.8%)	-	-	-	-
pT2	30 (23.3%)	-	-	-	-
pT3/T4	36 (27.9%)	-	-	-	-
Pathological grade, n (%)					
Low	37(28.7%)	-	-	-	-
High	92(71.3%)	-	-	-	-
Tumor diameter, n (%)					
<1cm	29 (22.5%)	-	-	-	-
1-3cm	74 (57.4%)	-	-	-	-
≧3cm	26 (20.1%)	-	-	-	-
Number of tumors, n (%)					
Single	48(37.2%)	-	-	-	-
Multiple	81(62.8%)	-	-	-	-
Surgical Methods					
TURBT	68(52.7%)	-	-	-	-
cystectomy	15(11.6%)	-	-	-	-
nephrouretectomy	46 (35.7%)	-	-	-	-
Lymphovascular invasion, n(%)					
Total	65	-	-	-	-
Positive	18 (27.70%)	-	-	-	-
Negative	47 (72.30%)	-	-	-	-
Lymph node metastasis					
Positive	10 (7.8%)	-	-	-	-
Negative	119 (92.2%)	-	-	-	-
Chemotherapy					
Intravesical	69 (53.5%)	-	-	-	-
adjuvant	32 (24.8%)	-	-	-	-
No	28 (21.7%)	-	-	-	-
Surgical margin, n (%)					
Positive	7 (5.4%)	-	-	-	-
Negative	122 (94.6%)	-	-	-	-

UC: Urothelial cancer; UTI: urinary tract infection; TURBT: transurethral resection of bladder tumor

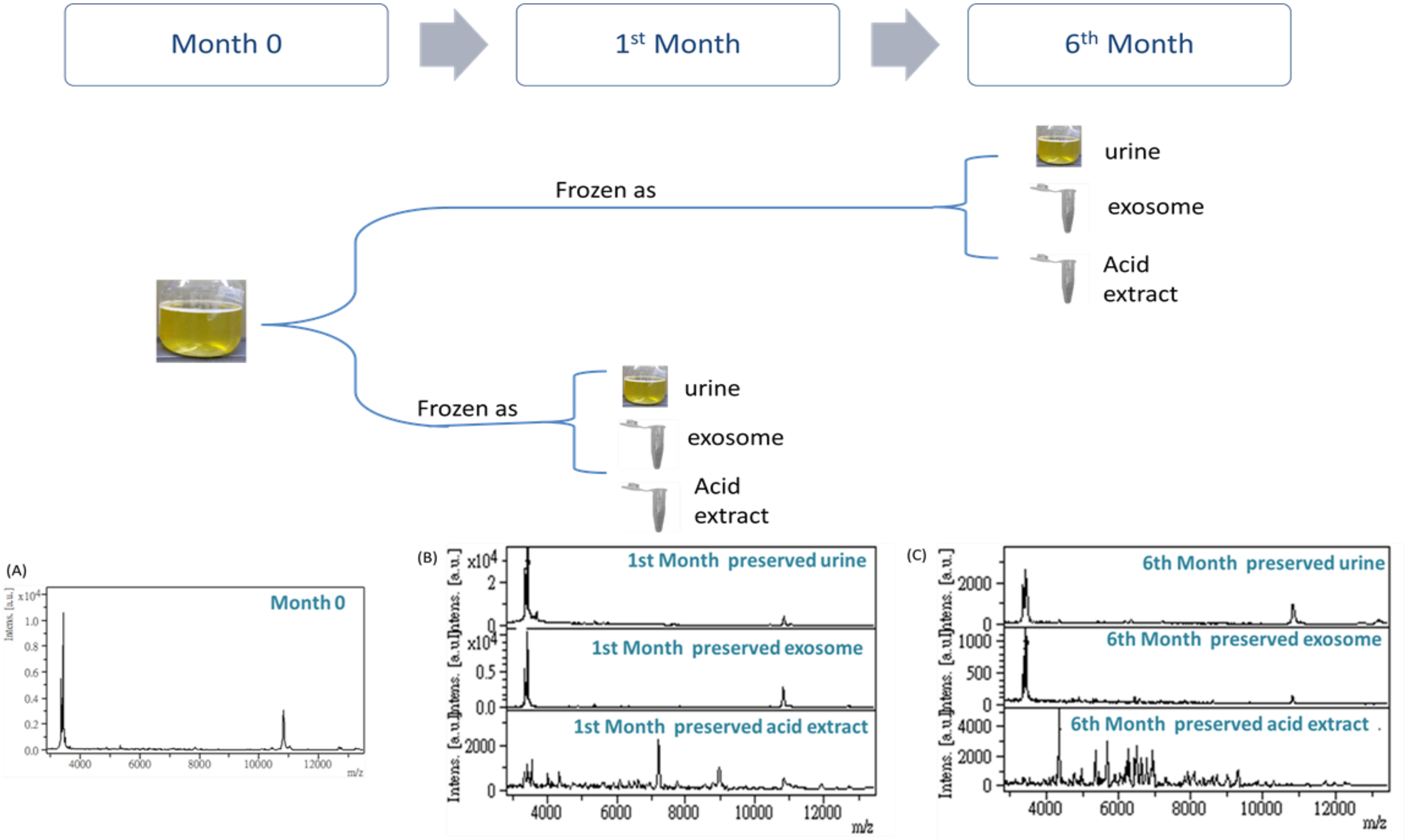

Supplemental Figure 1. MALDI-TOF spectra of urine exosomes stored as urine, exosomes, or acid extract at (A) Month 0, (B) first month, and (C) sixth month.

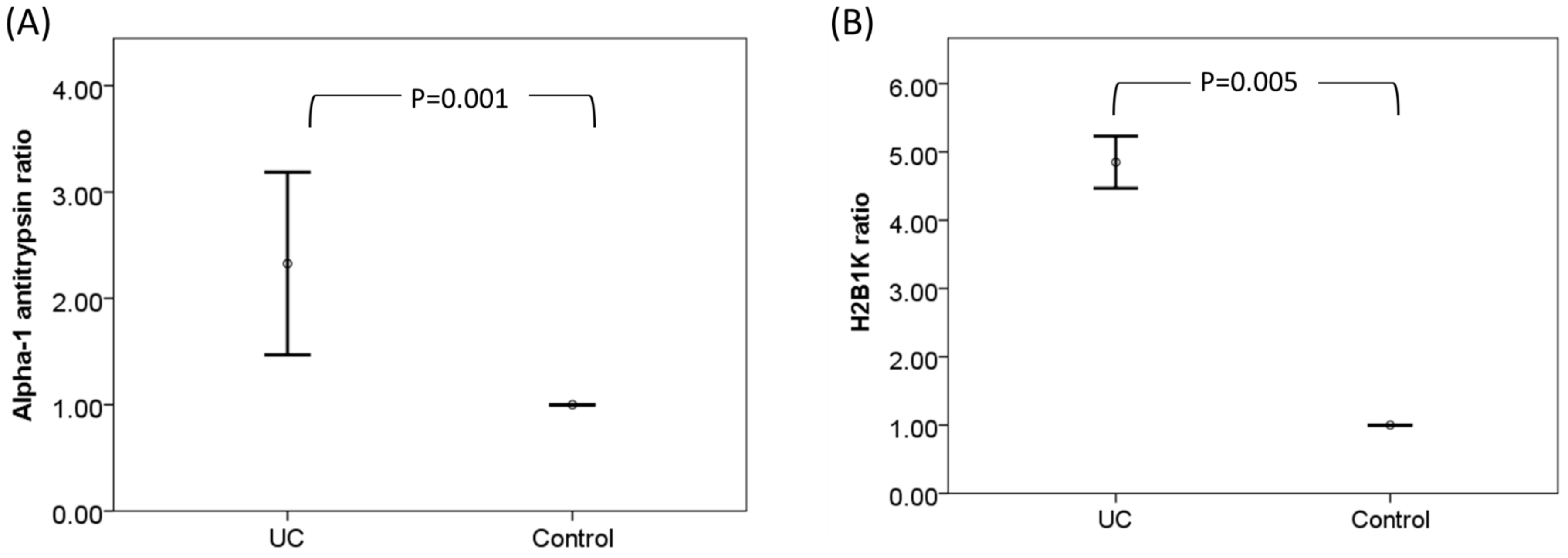

Supplemental Figure 2. Quantitative comparison of the urinary exosome proteins (A) alpha 1-antitrypsin and (B) H2B1K between UC and control group

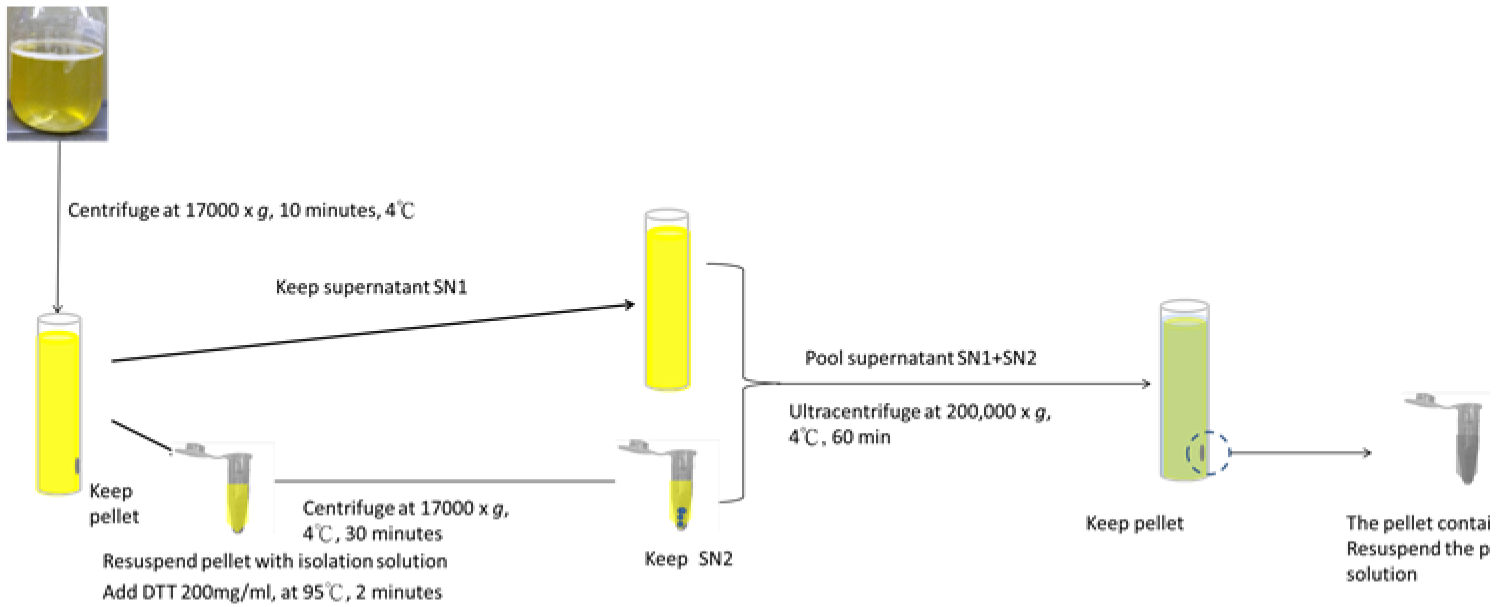

Supplemental Figure 3. Protocol for isolating urinary microparticles.

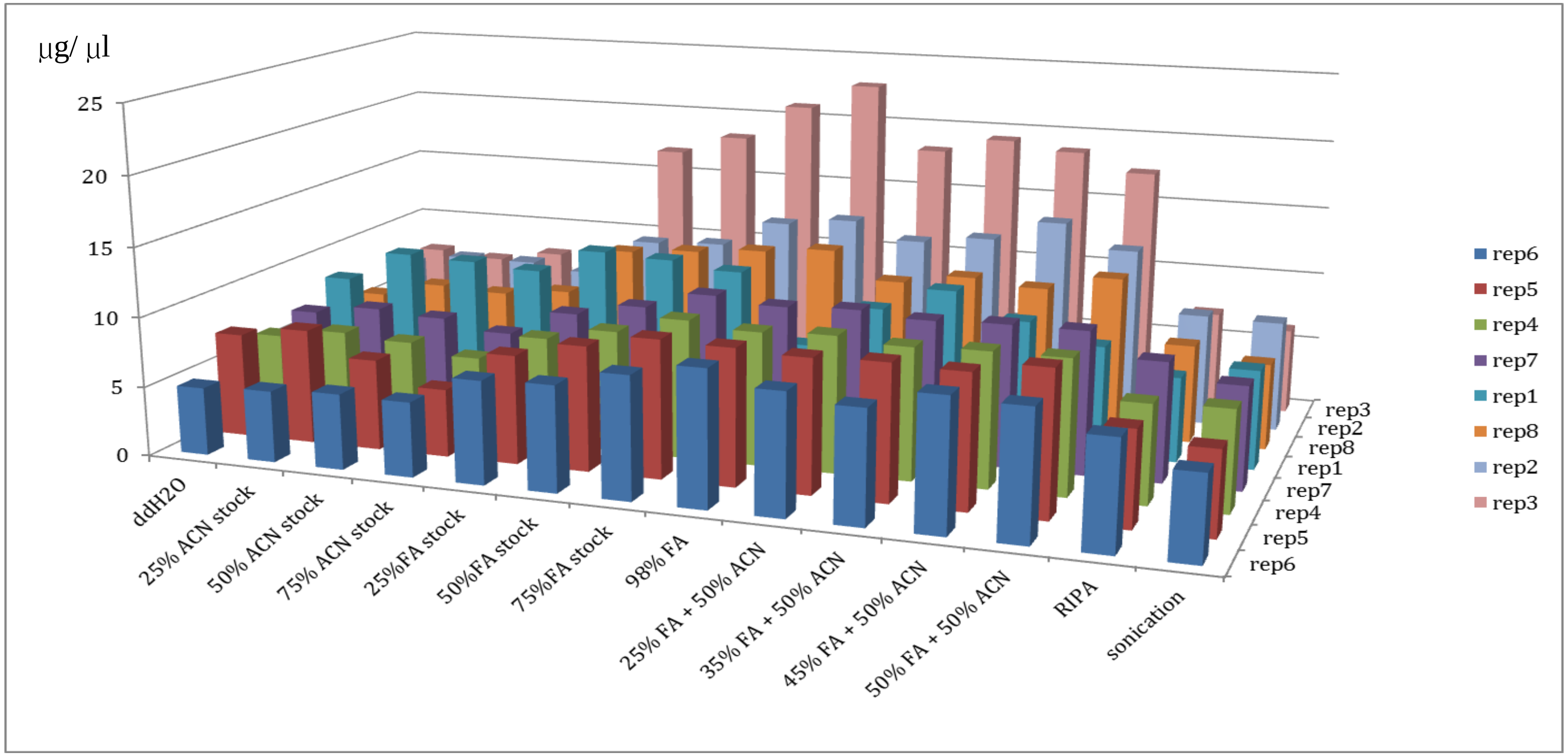

Supplemental Figure 4

This three-dimensional axis plot characterizes the biological replicates and technique replicates for evaluating extraction procedures: X axis represents 14 different extraction methods, Y axis is the concentration of extracted protein (μg/μl), and Z axis is the biological replicate.

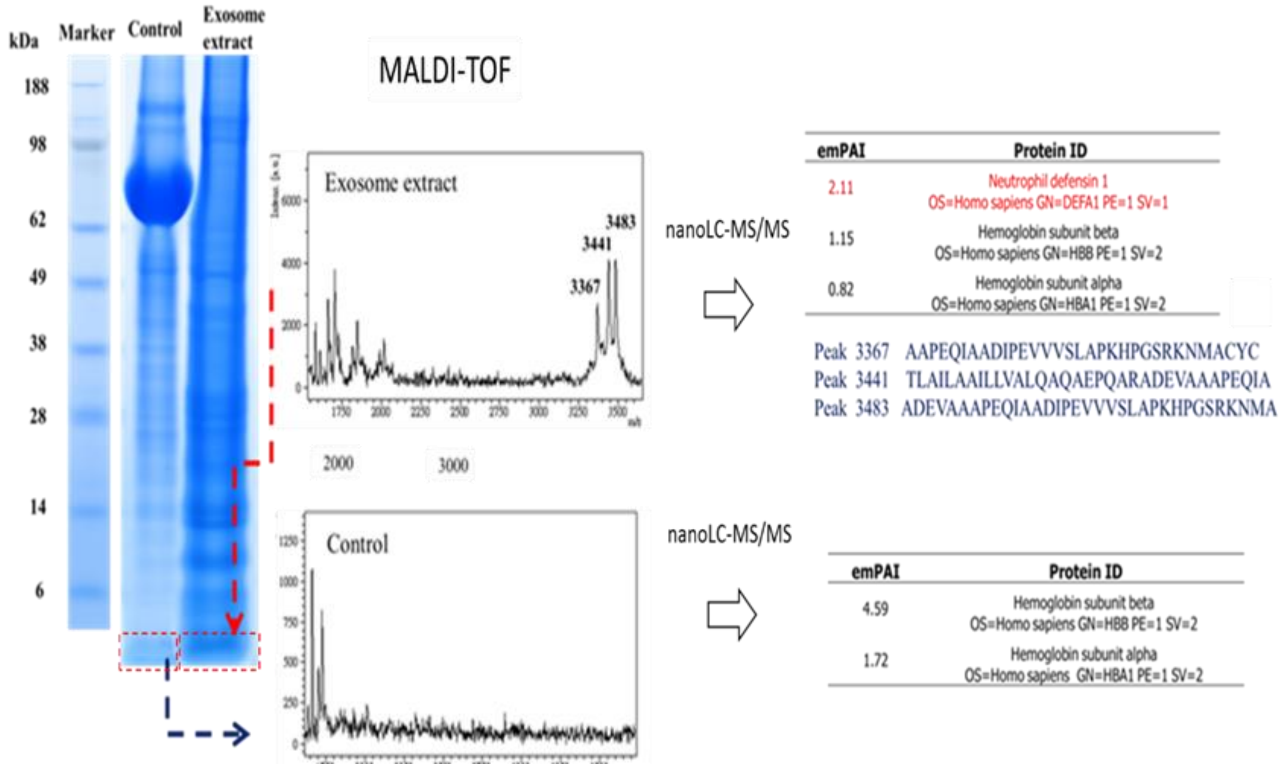

Supplemental Figure 5 The exosomes proteins and controls were both separated on 4%–12% Bis-Tris gels. Peptide bands of approximately 3000 Da were excised and extracted using 50% ACN/0.1% FA. The extracted peptides were analyzed through MALDI–TOF spectrometry to confirm the presence of peaks at m/z 3367, m/z 3441, and m/z 3483, which were only detected in peptide bands from exosome extracts. Neutrophil defensin 1 was only identified in the excised peptide band from exosome extracts, the peptide fragments of Neutrophil Defensin 1 can also match with their detected mass on MALDI–TOF.
